# Supplementary material for: DNA Double-Strand Break Resection Occurs during Non-homologous End Joining in G1 but Is Distinct from Resection during Homologous Recombination
Source: Mol Cell. 2017 Feb 16;65(4):671–684.e5. doi: 10.1016/j.molcel.2016.12.016 (PMC5316416; doi:10.1016/j.molcel.2016.12.016)
Supplement: Document S1. Figures S1–S7 and Tables S1 and S2 [file mmc1.pdf]

Molecular Cell, Volume *65*

Supplemental Information

DNA Double-Strand Break Resection Occurs during Non-homologous End Joining in G1 but Is Distinct from Resection during Homologous Recombination

Ronja Biehs, Monika Steinlage, Olivia Barton, Szilvia Juhász, Julia Künzel, Julian Spies, Atsushi Shibata, Penny A. Jeggo, and Markus Löbrich

Figure S1

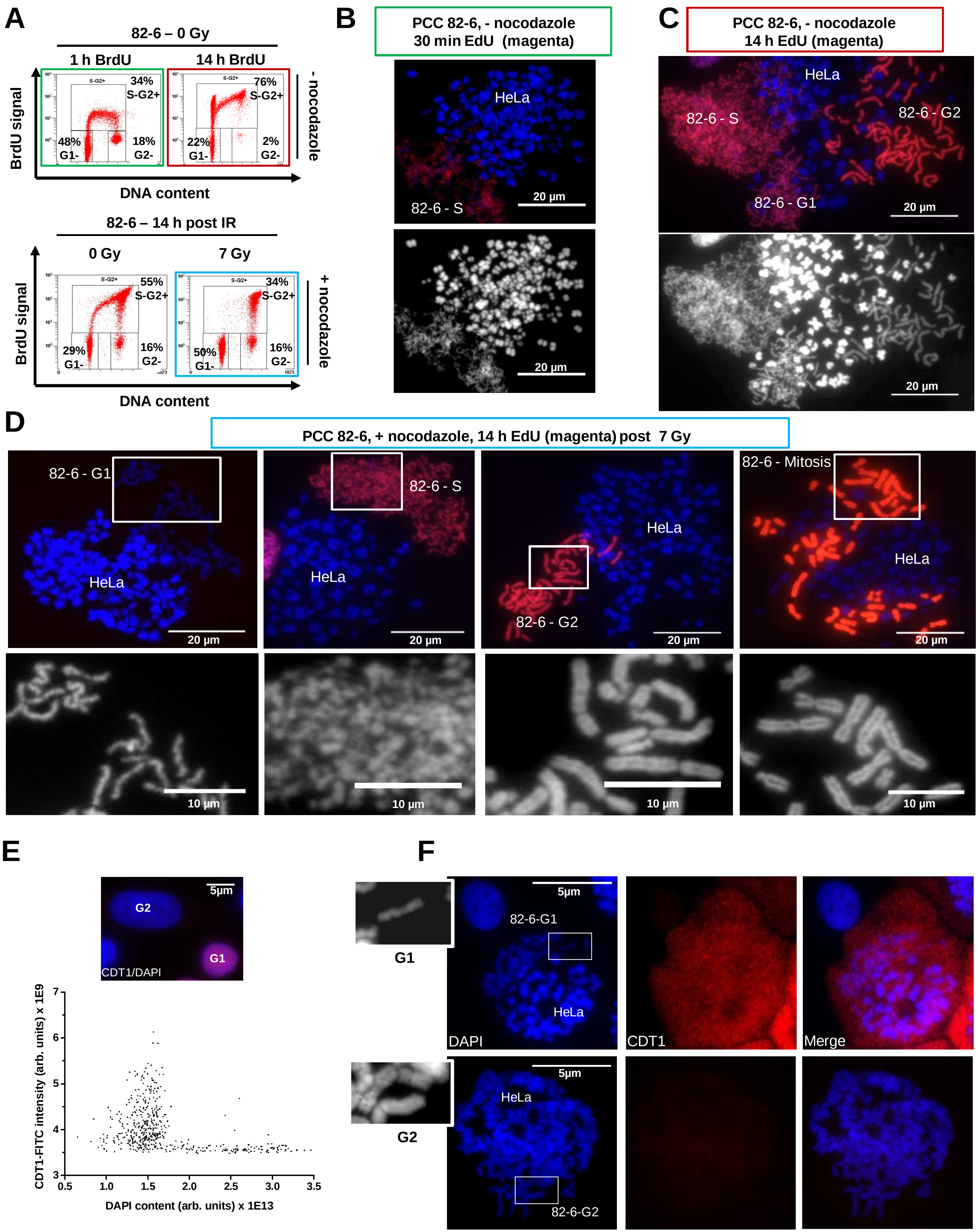
A
82-6 – 0 Gy
1 h BrdU
14 h BrdU
BrdU signal
34% S-G2+
48% G1-
18% G2-
76% S-G2+
22% G1-
2% G2-
- nocodazole
DNA content
82-6 – 14 h post IR
0 Gy
7 Gy
55% S-G2+
29% G1-
16% G2-
34% S-G2+
50% G1-
16% G2-
+ nocodazole
B
PCC 82-6, - nocodazole
30 min EdU (magenta)
HeLa
82-6 - S
20 µm
C
PCC 82-6, - nocodazole
14 h EdU (magenta)
HeLa
82-6 - S
82-6 - G2
82-6 - G1
D
PCC 82-6, + nocodazole, 14 h EdU (magenta) post 7 Gy
82-6 - G1
82-6 - S
82-6 - G2
82-6 - Mitosis
10 µm
E
G2
G1
5µm
CDT1/DAPI
CDT1-FITC intensity (arb. units) x 1E9
DAPI content (arb. units) x 1E13
0.5
1.0
1.5
2.0
2.5
3.0
3.5
F
82-6-G1
82-6-G2
DAPI
CDT1
Merge

Figure S2

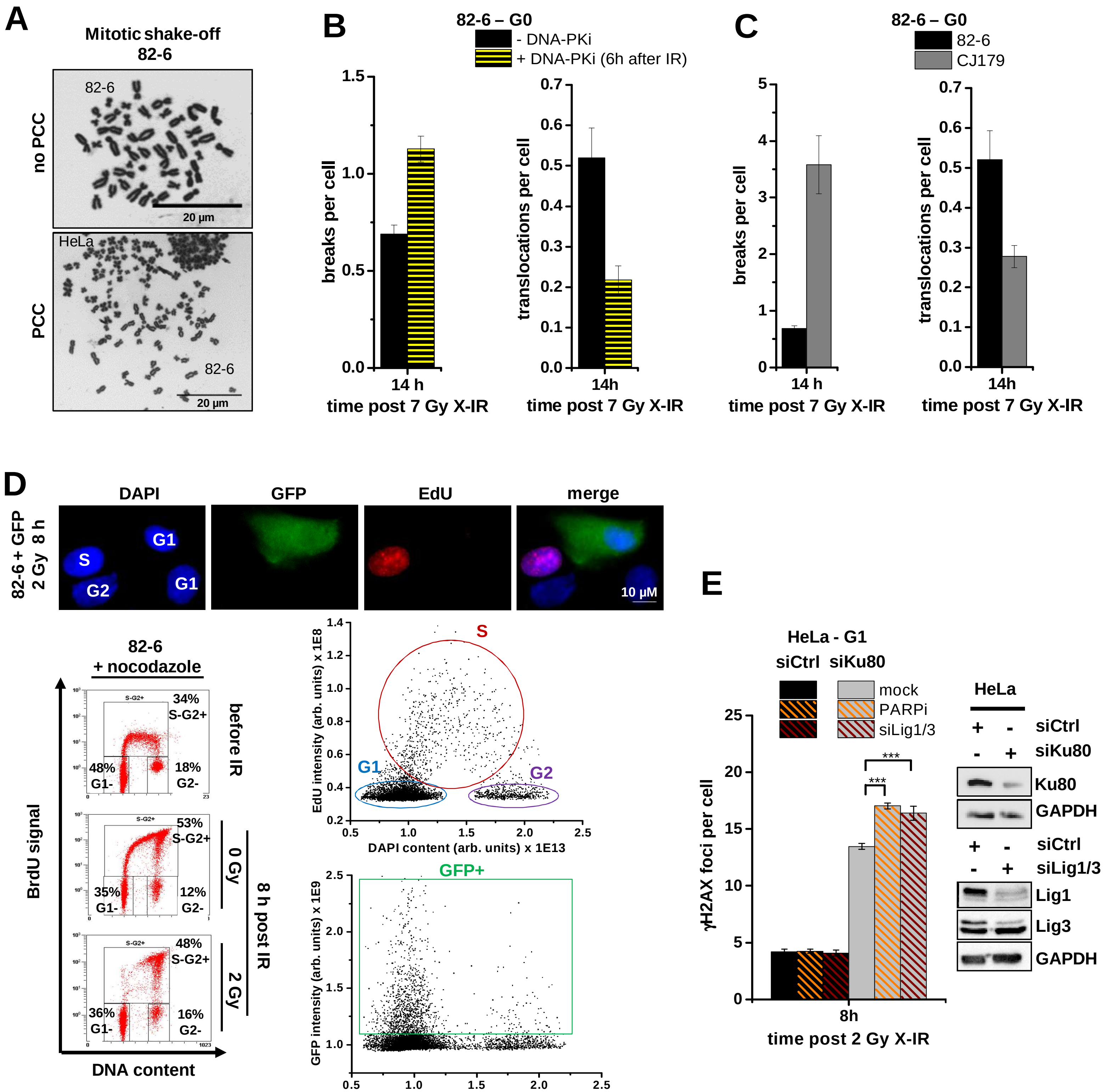
A
Mitotic shake-off
82-6
no PCC
PCC
82-6
HeLa
20 µm
B
82-6 – G0
- DNA-PKi
+ DNA-PKi (6h after IR)
breaks per cell
translocations per cell
14 h
14h
time post 7 Gy X-IR
C
82-6 – G0
82-6
CJ179
D
DAPI
GFP
EdU
merge
82-6 + GFP 2 Gy 8 h
S
G1
G2
10 µM
82-6
+ nocodazole
BrdU signal
DNA content
before IR
0 Gy
2 Gy
8 h post IR
34% S-G2+
48% G1-
18% G2-
53% S-G2+
35% G1-
12% G2-
48% S-G2+
36% G1-
16% G2-
EdU intensity (arb. units) x 1E8
DAPI content (arb. units) x 1E13
GFP+
GFP intensity (arb. units) x 1E9
E
HeLa - G1
siCtrl siKu80
mock
PARPi
siLig1/3
γH2AX foci per cell
8h
time post 2 Gy X-IR
HeLa
siCtrl
siKu80
Ku80
GAPDH
siLig1/3
Lig1
Lig3

Figure S3

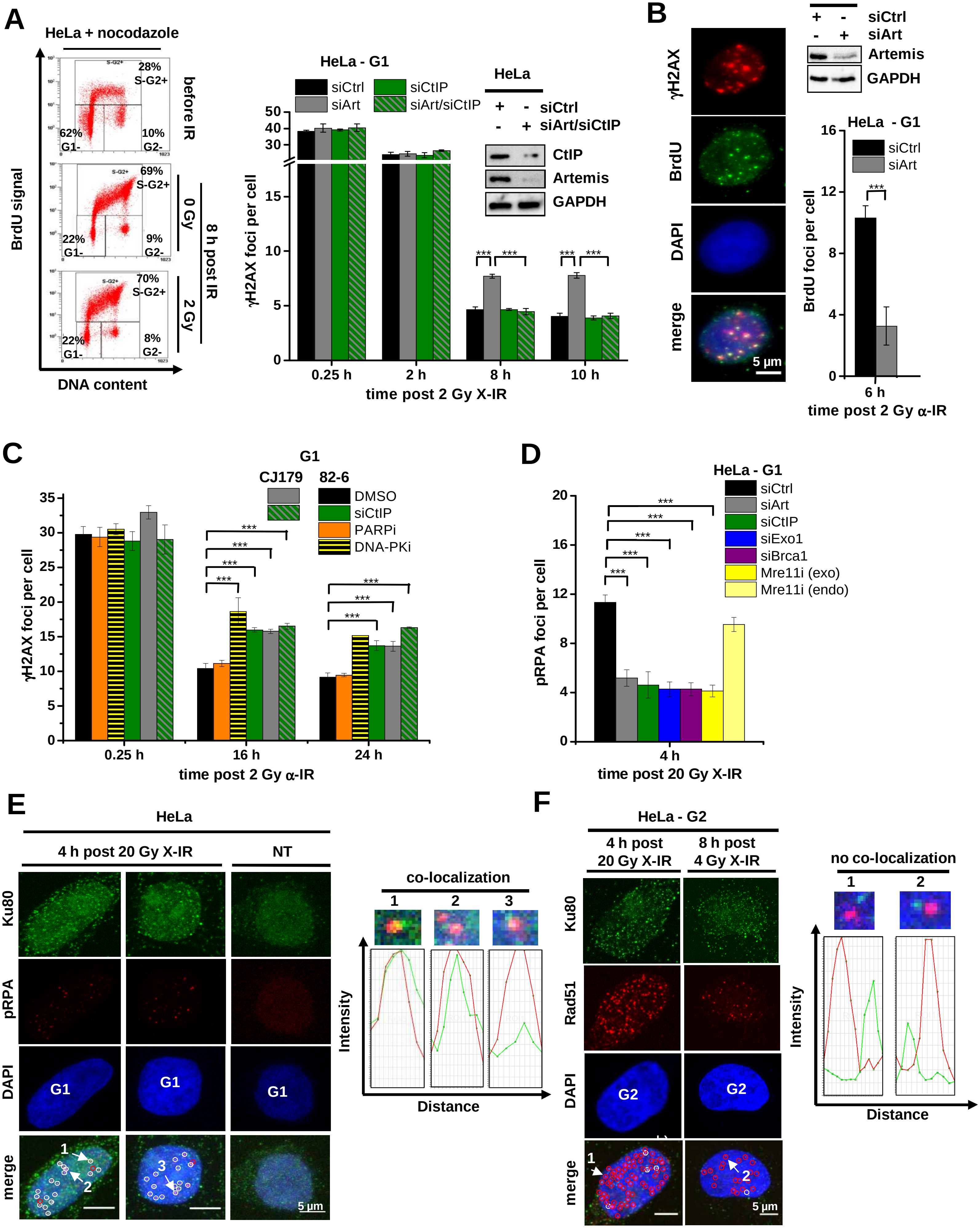
A
HeLa + nocodazole
BrdU signal
DNA content
before IR
0 Gy
2 Gy
8 h post IR
28% S-G2+
62% G1-
10% G2-
69% S-G2+
22% G1-
9% G2-
70% S-G2+
22% G1-
8% G2-
HeLa - G1
siCtrl
siArt
siCtIP
siArt/siCtIP
γH2AX foci per cell
0.25 h
2 h
8 h
10 h
time post 2 Gy X-IR
HeLa
siCtrl
siArt/siCtIP
CtIP
Artemis
GAPDH
B
γH2AX
BrdU
DAPI
merge
5 µm
HeLa
siCtrl
siArt
Artemis
GAPDH
HeLa - G1
BrdU foci per cell
6 h
time post 2 Gy α-IR
C
G1
CJ179
82-6
DMSO
siCtIP
PARPi
DNA-PKi
γH2AX foci per cell
0.25 h
16 h
24 h
time post 2 Gy α-IR
D
HeLa - G1
siCtrl
siArt
siCtIP
siExo1
siBrca1
Mre11i (exo)
Mre11i (endo)
pRPA foci per cell
4 h
time post 20 Gy X-IR
E
HeLa
4 h post 20 Gy X-IR
NT
Ku80
pRPA
DAPI
merge
G1
5 µm
co-localization
Intensity
Distance
F
HeLa - G2
4 h post 20 Gy X-IR
8 h post 4 Gy X-IR
Ku80
Rad51
DAPI
merge
G2
5 µm
no co-localization
Intensity
Distance

Figure S4

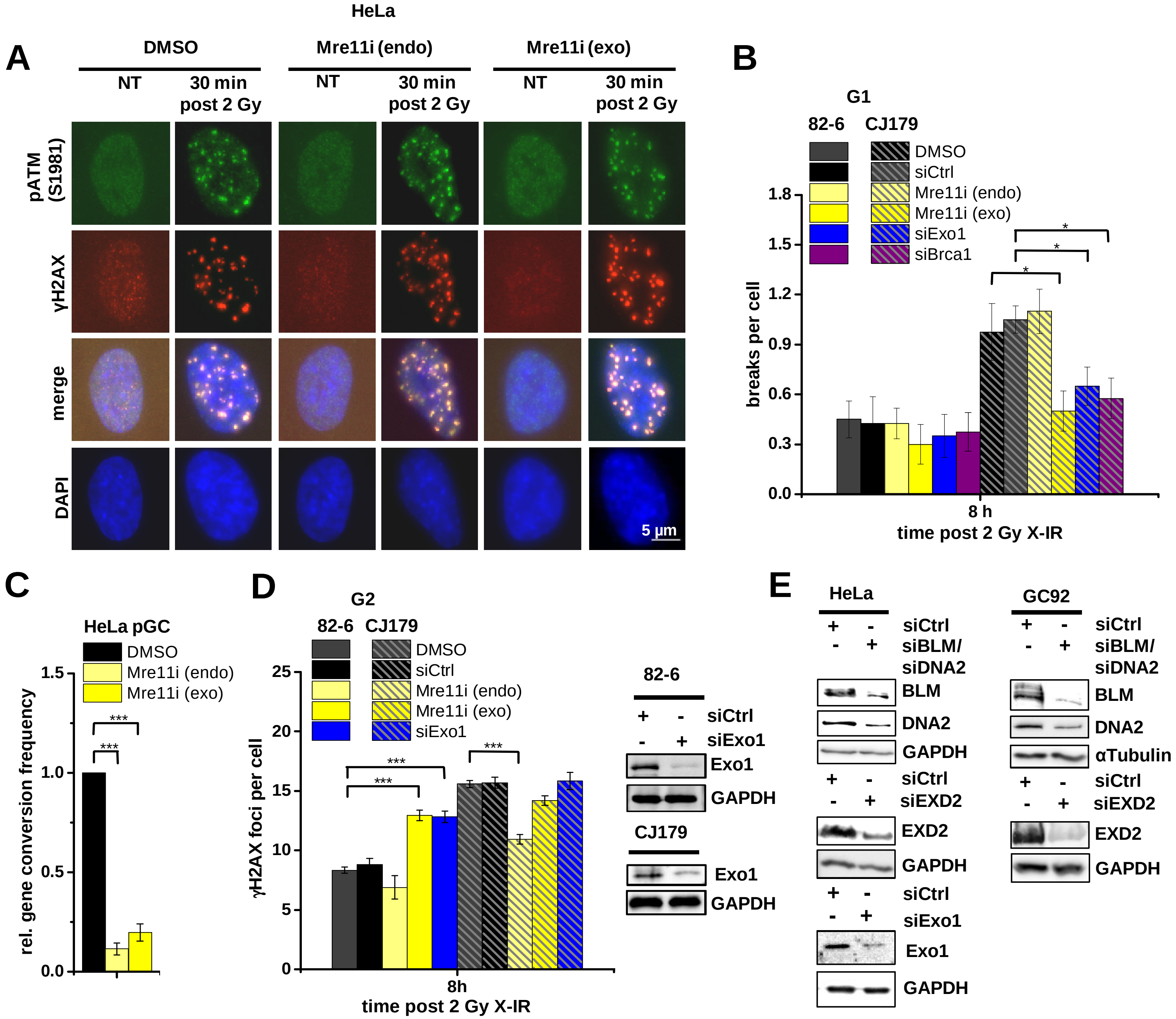

HeLa
A
DMSO
Mre11i (endo)
Mre11i (exo)
NT
30 min post 2 Gy
pATM (S1981)
γH2AX
merge
DAPI
5 µm
B
G1
82-6
CJ179
DMSO
siCtrl
Mre11i (endo)
Mre11i (exo)
siExo1
siBrca1
breaks per cell
0.0
0.3
0.6
0.9
1.2
1.5
1.8
*
8 h
time post 2 Gy X-IR
C
HeLa pGC
DMSO
Mre11i (endo)
Mre11i (exo)
rel. gene conversion frequency
0.0
0.5
1.0
1.5
***
D
G2
82-6
CJ179
DMSO
siCtrl
Mre11i (endo)
Mre11i (exo)
siExo1
γH2AX foci per cell
0
5
10
15
20
25
***
8h
time post 2 Gy X-IR
82-6
siCtrl
siExo1
Exo1
GAPDH
CJ179
Exo1
GAPDH
E
HeLa
siCtrl
siBLM/ siDNA2
BLM
DNA2
GAPDH
siCtrl
siEXD2
EXD2
GAPDH
siCtrl
siExo1
Exo1
GAPDH
GC92
siCtrl
siBLM/ siDNA2
BLM
DNA2
αTubulin
siCtrl
siEXD2
EXD2
GAPDH

Figure S5

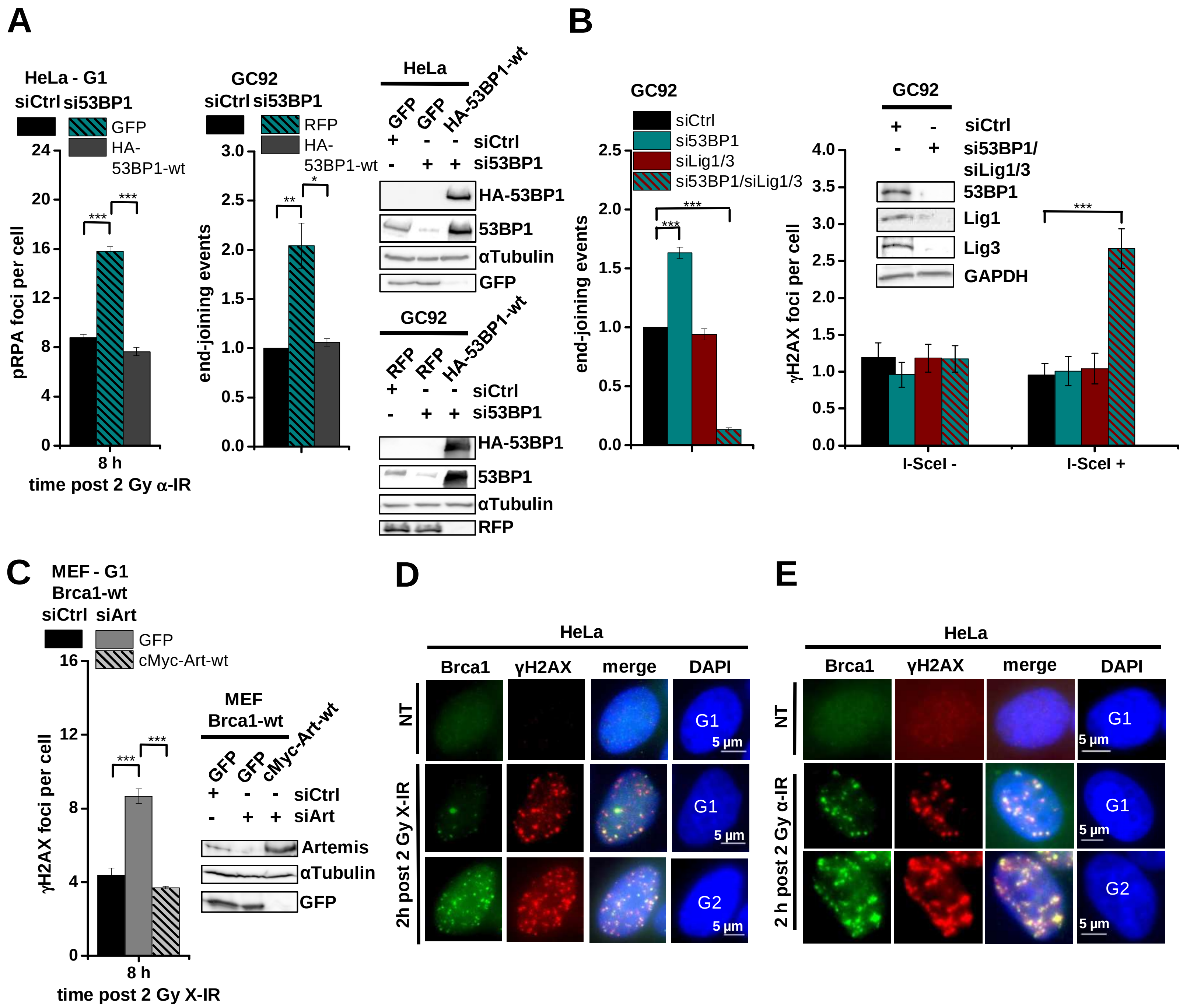
A
HeLa - G1
siCtrl si53BP1
GFP
HA-53BP1-wt
pRPA foci per cell
8 h
time post 2 Gy α-IR
GC92
siCtrl si53BP1
RFP
HA-53BP1-wt
end-joining events
HeLa
GFP GFP HA-53BP1-wt
siCtrl
si53BP1
HA-53BP1
53BP1
αTubulin
GFP
GC92
RFP RFP HA-53BP1-wt
siCtrl
si53BP1
HA-53BP1
53BP1
αTubulin
RFP
B
GC92
siCtrl
si53BP1
siLig1/3
si53BP1/siLig1/3
end-joining events
GC92
siCtrl
si53BP1/siLig1/3
53BP1
Lig1
Lig3
GAPDH
γH2AX foci per cell
I-SceI -
I-SceI +
C
MEF - G1
Brca1-wt
siCtrl siArt
GFP
cMyc-Art-wt
γH2AX foci per cell
8 h
time post 2 Gy X-IR
MEF
Brca1-wt
GFP GFP cMyc-Art-wt
siCtrl
siArt
Artemis
αTubulin
GFP
D
HeLa
Brca1
γH2AX
merge
DAPI
NT
2h post 2 Gy X-IR
G1
G2
5 µm
E
HeLa
Brca1
γH2AX
merge
DAPI
NT
2 h post 2 Gy α-IR
G1
G2
5 µm

Figure S6

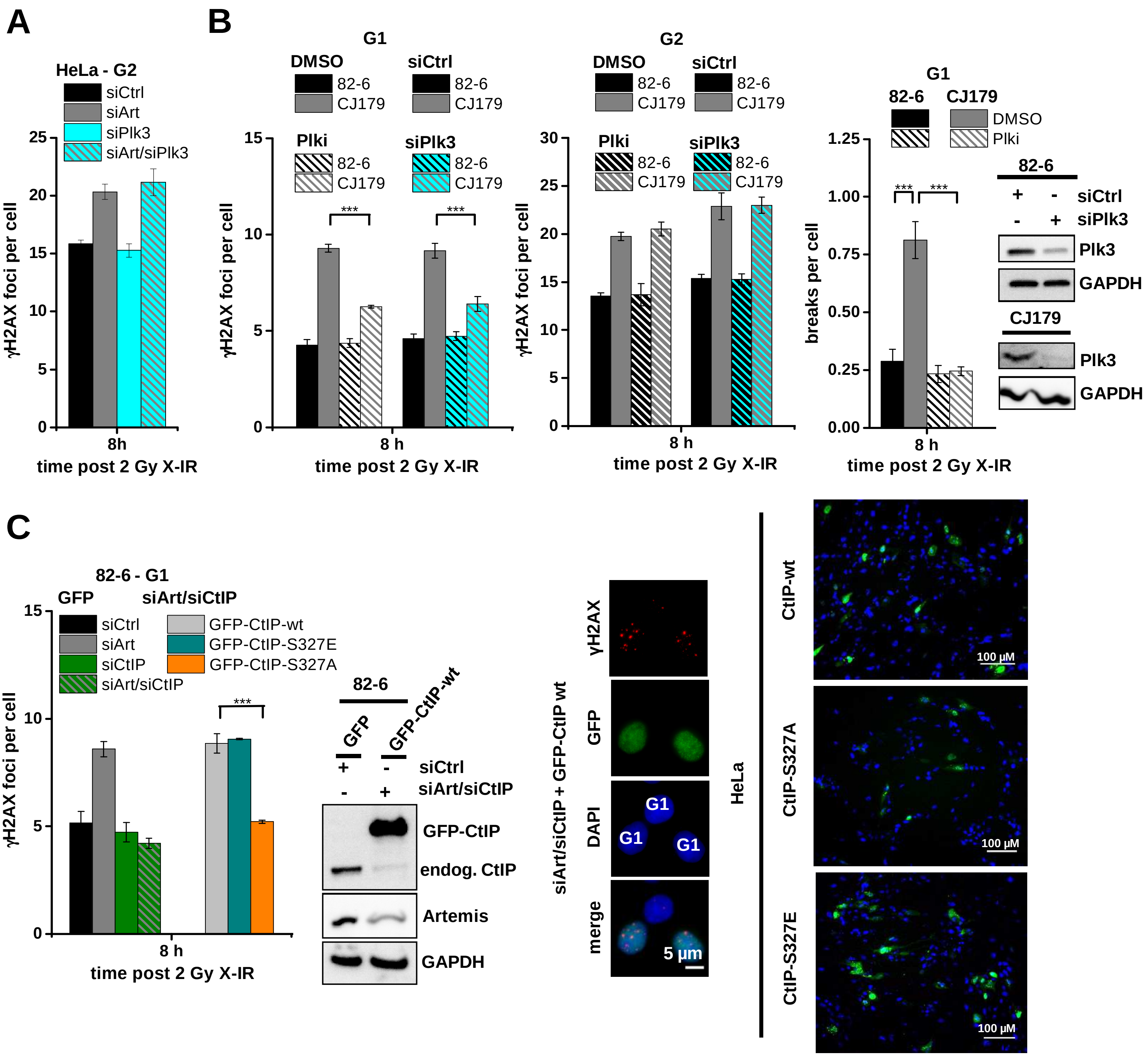
A
HeLa - G2
siCtrl
siArt
siPlk3
siArt/siPlk3
γH2AX foci per cell
8h
time post 2 Gy X-IR
B
G1
DMSO
siCtrl
82-6
CJ179
Plki
siPlk3
γH2AX foci per cell
8 h
time post 2 Gy X-IR
G2
breaks per cell
DMSO
Plki
siCtrl
siPlk3
Plk3
GAPDH
C
82-6 - G1
GFP
siArt/siCtIP
siCtrl
siArt
siCtIP
siArt/siCtIP
GFP-CtIP-wt
GFP-CtIP-S327E
GFP-CtIP-S327A
82-6
GFP
GFP-CtIP-wt
siCtrl
siArt/siCtIP
GFP-CtIP
endog. CtIP
Artemis
GAPDH
siArt/siCtIP + GFP-CtIP wt
γH2AX
GFP
DAPI
merge
G1
5 µm
HeLa
CtIP-wt
CtIP-S327A
CtIP-S327E
100 µM

Figure S7

A

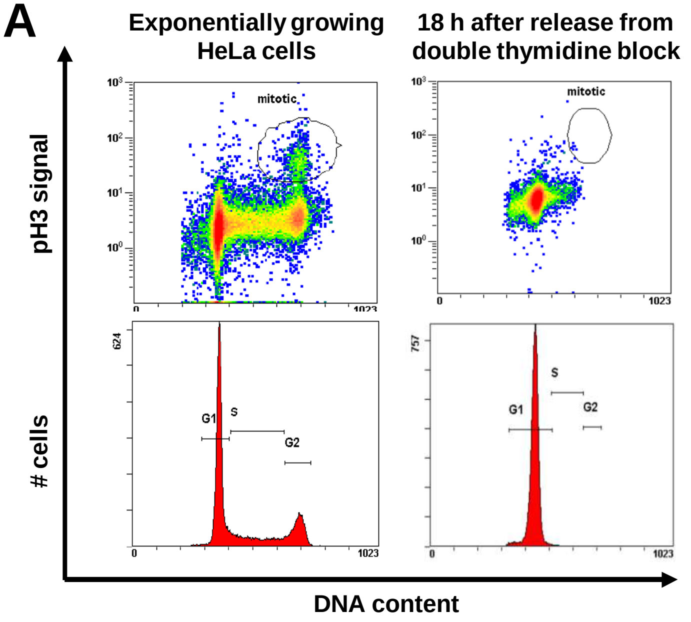

B

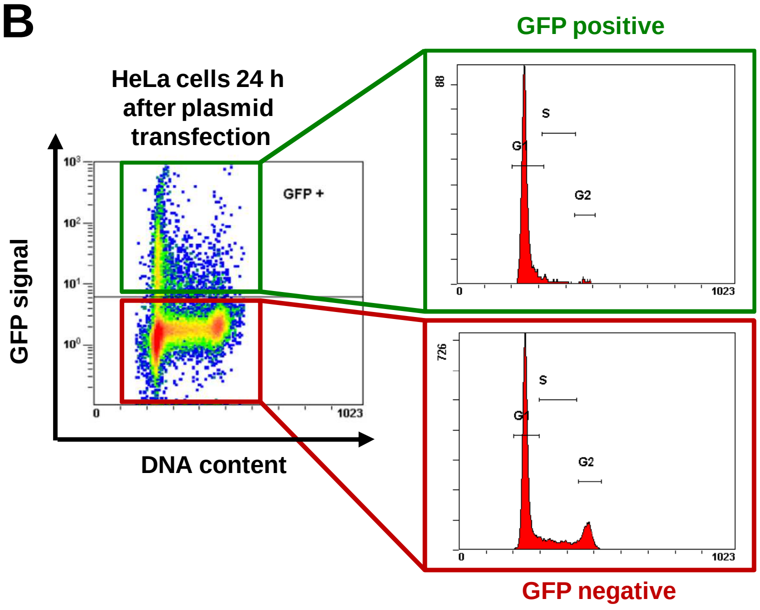

C

Model for processing of resection intermediates

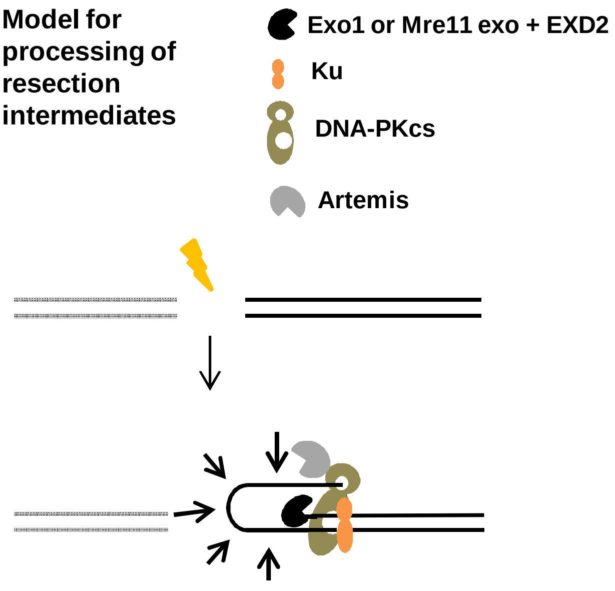

SUPPLEMENTAL FIGURE LEGENDS

Figure S1. Related to Figure 1

(A) Upper images: Growing 82-6 cells were incubated with BrdU in the absence of nocodazole for 1 or 14 h before analysis by flow cytometry. Growth in the presence of BrdU (and absence of nocodazole) for 14 h results in $BrdU^+$ G1 cells and depletion of the $BrdU^-$ G2 population. Lower images: 82-6 cells were treated with nocodazole, incubated with BrdU, irradiated or not, and analyzed 14 h later. Nocodazole efficiently prevented cells from progressing into G1 as evidenced by the absence of $BrdU^+$ G1 cells and the maintenance of the $BrdU^-$ G2 population. IR resulted in efficient G1 checkpoint induction as evidenced by the absence of early- and mid-S phase cells.

(B) Growing unirradiated 82-6 cells were pulse-labeled with EdU for 30 min and analyzed by PCC. Heavily damaged chromosome structures were confined to EdU^+ S-phase cells.

(C) Growing 82-6 cells were incubated with EdU, left unirradiated, not treated with nocodazole and analyzed by PCC 14 h later. Since cells progressed through the cell cycle uninhibited, EdU^+ chromosome spreads were obtained in heavily damaged S-phase cells as well as in G1 cells with a one-chromatid and in G2 cells with a two-chromatid morphology.

(D) Growing 82-6 cells were incubated with EdU, irradiated, treated with nocodazole and analyzed by PCC 14 h later. Since nocodazole prevented progression from G2 to G1, all cells with a one-chromatid morphology were negative for EdU whilst EdU^+ cells were frequently detected in spreads with heavily damaged chromosomes and in spreads with a two-chromatid morphology. Two-chromatid G2 and mitotic cells can be easily distinguished from one-chromatid G1 cells.

(E) Growing HeLa cells were stained for the G1 marker CDT1 (upper panel) and the CDT1 signal was plotted as a function of the DAPI signal (lower panel). Only G1 cells stain positive for CDT1.

(F) Growing 82-6 cells were analyzed by PCC and stained against CDT1. The images show a mitotic HeLa cell fused with either a one-chromatid (upper row) or a two-chromatid 82-6 cell (lower row). Only the one-chromatid but not the two-chromatid PCC spread stained positive for the G1 marker CDT1 (the same was observed for all other one-chromatid and two-chromatid PCC spreads). Note that during cell rupture, which occurs while transferring the chromosomes to the glass slides, chromatin-bound CDT1 detaches from the chromosomes but remains detectable by IF staining in the nuclear area.

Figure S2. Related to Figure 1

(A) Chromosome spreads of mitotic 82-6 cells. Growing cells were incubated with nocodazole for 14 h, the mitotic cell fraction was harvested by mitotic shake-off and analyzed with or without the PCC approach. Both approaches failed to provide one-chromatid spreads while two-chromatid mitotic structures were clearly visible which typically are more condensed than the two-chromatid structures of G2 cells. This control experiment shows that mitotic 82-6 cells do not give rise to one-chromatid PCC spreads.

(B) Chromosome breaks and translocations in one-chromatid PCC spreads obtained from confluent 82-6 cells treated with DNA-PKi at 6 h post IR. The results confirm the analysis of the one-chromatid PCC spreads obtained from growing 82-6 cells in Figure 1D. Mean +/- SEM.

(C) Chromosome breaks and translocations in PCC spreads obtained from confluent 82-6 and Artemis-deficient CJ179 cells. The results confirm the analysis of the one-chromatid PCC spreads obtained from growing 82-6 and CJ179 cells in Figure 1E. Mean +/- SEM.

(D) Left panel: 82-6 cells were treated with nocodazole, incubated with BrdU, irradiated or not, and analyzed 8 h later by flow cytometry. Nocodazole efficiently prevented cells from progressing into G1 as evidenced by the absence of $BrdU^+$ G1 cells and the maintenance of the $BrdU^-$ G2 population. IR resulted in efficient G1 checkpoint induction as evidenced by the absence of early S-phase cells. Right panels: Identification of cell cycle phases using a semi-automated scanning system. Cells were incubated with nocodazole and EdU 30 min prior to IR and during the entire repair period. Cells were scanned under the microscope; EdU and GFP signals were plotted against the DAPI signal. All EdU^+ S-phase cells were excluded from the analysis and only EdU^- G1 and G2 cells, which remained in these cell cycle phases during repair incubation, were analyzed. For complementation experiments, only GFP^+ EdU^- G1 cells were analyzed.

(E) γH2AX foci in G1 HeLa cells treated with siKu80 plus PARPi or siLig1/3. Mean +/- SEM.

Figure S3. Related to Figure 2

(A) Left panel: HeLa cells were treated with nocodazole, incubated with BrdU, irradiated or not, and analyzed 8 h later by flow cytometry. Nocodazole efficiently prevented cells from progressing into G1 as evidenced by the absence of $BrdU^+$ G1 cells and the maintenance of the $BrdU^-$ G2 population. Despite the lack of an efficient G1 checkpoint, a substantial proportion of cells remained in G1 during repair incubation. Right panel: γH2AX foci in G1 HeLa cells treated with siArtemis and/or siCtIP. Mean +/- SEM.

(B) BrdU foci in G1 HeLa cells treated with siArtemis. Mean +/- SEM.

(C) γH2AX foci in G1 82-6 and CJ179 cells treated with siCtIP, PARP or DNA-PK inhibitors. Mean +/- SEM.

(D) pRPA foci in G1 HeLa cells treated with siArtemis, siCtIP, siExo1, siBrca1 or Mre11 exo- or endonuclease inhibitors. Mean +/- SEM.

(E) Ku and pRPA staining in G1 HeLa cells. Co-localization was analyzed by line blots and observed in >95% of pRPA foci. White circles: Co-localizing foci; red circles: non-co-localizing foci.

(F) Ku and Rad51 staining in G2 HeLa cells. Co-localization was analyzed by line blots and observed in <20% (after 20 Gy, 4 h) or <10% (after 4 Gy, 8 h) of Rad51 foci. White circles: Co-localizing foci; red circles: non-co-localizing foci.

Figure S4. Related to Figure 4

(A) pATM and γH2AX foci in HeLa cells treated with Mre11 endo- or exonuclease inhibitor. NT: not treated.

(B) Chromosome breaks in G1 82-6 and CJ179 cells treated with Mre11 endo- or exonuclease inhibitor, siExo1 or siBrca1. Mean +/- SEM.

(C) Relative gene conversion frequencies in HeLa pGC cells containing an HR reporter substrate (Mansour et al., 2008) and treated with Mre11 endo- or exonuclease inhibitor. Mean +/- SEM.

(D) γH2AX foci in G2 82-6 and CJ179 cells treated with Mre11 endo- or exonuclease inhibitor or siExo1. Mean +/- SEM.

(E) Knock-down efficiencies in HeLa and GC92 cells.

Figure S5. Related to Figure 5

(A) pRPA foci in G1 HeLa and end-joining events in GC92 cells treated with si53BP1. Cells were transfected with GFP, RFP or HA-53BP1-wt constructs and GFP^+, RFP^+ or HA^+ cells were analyzed. Mean +/- SEM.

(B) Left panel: End-joining events in GC92 cells treated with siLig1/3 and/or si53BP1. Right panel: γH2AX foci in GC92 cells treated with siLig1/3 and/or si53BP1. Cells were transfected with I-SceI and foci were scored in I-$SceI^+$ and I-$SceI^-$ cells (identified by IF against I-SceI). Mean +/- SEM.

(C) γH2AX foci in G1 MEFs treated with siArtemis. Cells were transfected with GFP or cMyc-Artemis-wt constructs and foci were analyzed in GFP^+ or $cMyc^+$ G1 cells. Mean +/- SEM.

(D) and (E) Brca1 and γH2AX foci in G1 and G2 HeLa cells after X-IR (panel D) or α-IR (panel E). NT: not treated.

Figure S6. Related to Figure 6

(A) γH2AX foci in G2 HeLa cells treated with siArtemis and/or siPlk3. Mean +/- SEM.

(B) γH2AX foci and chromosome breaks in G1 and/or G2 82-6 and CJ179 cells treated with siPlk3 or Plki. Mean +/- SEM.

(C) Left panel: γH2AX foci in G1 82-6 cells treated with siArtemis and/or siCtIP. Cells were transfected with GFP, GFP-CtIP-wt, a phospho-mimic (GFP-CtIP-S327E) or a non-phosphorylatable mutant (GFP-CtIP-S327A) and foci were analyzed in GFP^+ G1 cells. Right panel: IF images showing similar transfection efficiencies for the various CtIP constructs. Mean +/- SEM.

Figure S7. Related to Figure 7

(A) Flow cytometry analysis of HeLa cells growing exponentially or 18 h after release from a double thymidine block, showing synchronization in G1 phase.

(B) Flow cytometry analysis of HeLa cells 24 h after transfection with GFP-CtIP constructs. The cell cycle distribution shows G1 synchronisation of the GFP^+ cells.

(C) Speculative model for processing of resection intermediates: Ku translocates inwards and restricts the extent of resection by Exo1 or Mre11 exonuclease/ EXD2. The ssDNA tail is captured by the ssDNA channel of DNA-PKcs generating a hairpin-like structure which is cleaved by Artemis bound to DNA-PKcs (arrows indicate potential cleavage sites). In this model Artemis is required for processing of trapped resection intermediates and subsequent formation of ssDNA. pRPA foci arise when cleavage results in a ssDNA tail long enough for RPA binding (estimated to be >20 bp). DNA-PKcs may either remain loosely bound to Ku80 during the entire process or may attach to the ss/dsDNA transition at the resected end after Ku translocation but before cleavage by Artemis.

Table S1. Sequence analysis of end-joining events after Lig1/3 depletion, related to Figure 3

Amount n=33	siCtrl + I-SceI	Size of deletion	Size of insertion
6 x	C A C G G A A G G A A T T A C C C T G T T A T C C C T A T	0	0
1 x	C A C G G A A G G A A T T A C C C T G T T - - C C C T A T	2	0
1 x	C A C G G A A G G A A T T A C C C T - - - A T C C C T A T	3	0
1 x	C A C G G A A G G A A T T A - - - T G T T A T C C C T A T	3	4
1 x	C A C G G A A G G A A T T A C C C T G - - - - C C C T A T	4	0
1 x	C A C - - - - G G A A T T A C C C T G T T A T C C C T A T	4	0
1 x	C A C G G A A G G A A T T A C C C T G T T - - - - - - A T	6	0
2 x	C A C G G A A G G A A T T A - - - - - - - - T C C C T A T	8	0
6 x	C A C G G A A G G A A T T A C C C T - - - - - - - - - A T	9	0
1 x	C A C G G A A G G A A T - - - - - - - - - - - - - - T A T	14	2
1 x	C A C G G A A G G - - - - - - - - - - - - - - - C C T A T	15	6
1 x	C A - C T A T	23	7
1 x	C G C T - [-] - - - - - - - - - - - - - - - - - C T A T	34	0
1 x	C G C T - [-] - - - - - - - - - - - - - - - - - C T A T	34	3
1 x	G C G C - [-] - - - - - - - - - - - - - - - - - - T A T	36	0
1 x	C A C G - - - - - - - - - - - - - - - - - [-] - C C A T	42	0
1 x	A G A G - [-] - - - - - - - - - - - - - [-] - T C A C	44	10
1 x	C T G G - [-] - - - - - - - - - - - - - [-] - C T A G	46	13
1 x	A A T A - [-] - T	73	0
1 x	C A C G - - - - - - - - - - - - - - - - - [-] - A G C T	109	0
1 x	C A C G G A - - - - - - - - - - - - - - - [-] - G C T G	266	2
1 x	C T T G - [-] - - - - - - - - - - - - - [-] - C G G T	517	0

Amount n=25	siLig1/3 + I-SceI	Size of deletion	Size of insertion
5 x	C A C G G A A G G A A T T A C C C T G T T A T C C C T A T	0	0
1 x	C A C G G A A G G A A T T A C C C T - T T A T C C C T A T	1	0
1 x	C A C G G A A G G A A T T A C C C T - T T A T C C C T A T	1	13
1 x	C A C G G A A G G A A T T A C C C T - - - A T C C C T A T	3	0
2 x	C A C G G A A G G A A T T A - - - - - - - - T C C C T A T	8	0
1 x	C A C G G A A G G A A T T - - - - - - - - - A T C C C T A T	8	0
1 x	C A C G G A A G G A A T - - - - - - - - - - A T C C C T A T	9	0
1 x	C A C G G A A G G A A T T A C C C T G T - [-] - C T A G	9	0
2 x	C A C G G A A G G A A T T A C C C T - - - - - - - - - A T	9	0
1 x	C A C G G A A G G A A - - - - - - - - - - - T C C C T A T	11	0
1 x	C A C G G A A G G A A T T A C C - - - - - - - - - C T A T	13	3
1 x	C A C G G A A G G A - - - - - - - - - - - [-] - T A T G	24	0

1 x	C A C G G A A - - - - - - - - - - - - - - [-] - T C A C	34	0
1 x	T A G A - [-] - - - - - - - - - - - - - [-] - T A T G	38	0
1 x	G C T A - [-] - T	42	0
1 x	T G G C - [-] - - - - - - - - - - - - - [-] - C A T G	63	0
1 x	C C A T - [-] - - - - - - - - - - - - - - - - - - - **A** T	174	0
1 x	C A - - - - - - - - - - - - - - - - - - - [-] - G C T G	281	0
1 x	C A C G G A A G G A **A** - - - - - - - - - - [-] - C T G C	342	0

Sequence analysis of GC92 wt and siLig1/3-treated cells. The 18 nucleotide long I-SceI recognition sequence is in **bold**, the characteristic I-SceI cutting site is labeled yellow, and microhomologies are labeled red. All sequences are arranged according to their siRNA treatment and to the size of their deletion. Some sequences contained inserted nucleotides in their deleted region.

List of Insertions

siCtrl

D=3; I=4 : TTCA

D=14; I=2 : CA

D=15; I=6 : GTAATT

D=23; I=7 : AGCTGTT

D=34; I=3 : TGG

D=44; I=10 : CACAACACGG

D=46; I=13 : GTAAGCTTACAAG

D=266; I=2 : CA

siLig1/3

D=1; I=13 : AGCTAGATATGAA

D=13; I=3 : TAT

Table S2. List of sequence-based reagents used in this study, related to Star Methods

Sequence-Based Reagents		
siRNA targeting sequence: human 53BP1:<br>AGA ACG AGG AGA CGG UAA UAG UGG G	Qiagen	N/A
siRNA targeting sequence: human 53BP1:<br>GAG AGC AGA TGA TCC TTT A	Dharmacon	N/A
siRNA targeting sequence: human Artemis:<br>AAC TGA AGA GAG CTA GAA CAG	Qiagen	N/A
siRNA targeting sequence: mouse Artemis:<br>AAG GAT CAC ATG AAA GGA TTA	Qiagen	N/A
siRNA targeting sequence: human BLM:<br>AAG CTA GGA GTC TGC GTG CGA	Qiagen	N/A
siRNA targeting sequence: human Brca1:<br>AAT CAC AGT GTC CTT TAT GTA	Qiagen	N/A
siRNA targeting sequence: human CtIP1:<br>TCC ACA ACA TAA TCC TAA TAA	Qiagen	N/A
siRNA targeting sequence: human CtIP2:<br>AAG CTA AAA CAG GAA CGA ATC	Qiagen	N/A
siRNA targeting sequence: human DNA2:<br>AAA TAG CCA GTA GTA TTC GAT	Qiagen	N/A
siRNA targeting sequence: human DNA-PKcs:<br>CTC GTG TAT TAC AGA AGG AAA	Qiagen	N/A
siRNA targeting sequence: human EXD2: smart pool	Dharmacon	N/A
siRNA targeting sequence: human Exo1:<br>CAA GCC TAT TCT CGT ATT TTT	Qiagen	N/A
siRNA targeting sequence: human Ku70:<br>GGA AGA GAT AGT TTG ATT TTT	Qiagen	N/A
siRNA targeting sequence: human Ku80:<br>AAG ACA GAC ACC CTT GAA GAC	Qiagen	N/A
siRNA targeting sequence: human Lig1:<br>GGC ATG ATC CTG AAG CAG A	Qiagen	N/A
siRNA targeting sequence: human Lig3:<br>CCA CAA AAA AAA TCG AGG A	Qiagen	N/A
siRNA targeting sequence: human Lig4:<br>CAA GAT GTT TAC AGA AAG GAA	Qiagen	N/A
siRNA targeting sequence: human Plk3:<br>CTG CAT CAA GCA GGT TCA CTA	Qiagen	N/A
siRNA targeting sequence: control:<br>AAT TCT CCG AAC GTG TCA CGT	Qiagen	N/A
Artemis gRNA sequence:<br>GAG ACT TCA GAT TGG CGC A<br>GAG CCC GTA CCA TGT TGT G	This paper	N/A
PCR primer sequence: CMV2:<br>ATA TAT GGA GTT CCG CGT TAC AT	This paper	Eurofins
PCR primer sequence: CD4int:<br>GCT GCC CCA GAA TCT TCC TCT	This paper	Eurofins

SiRNA targeting sequences used for protein downregulation, gRNA sequences used for generation of CRISPR/Cas9 knockout and PCR primer sequences used for sequence analysis.
