# Supplementary material for: DNA Double-Strand Break Resection Occurs during Non-homologous End Joining in G1 but Is Distinct from Resection during Homologous Recombination
Source: Mol Cell. 2017 Feb 16;65(4):671–684.e5. doi: 10.1016/j.molcel.2016.12.016 (PMC5316416; doi:10.1016/j.molcel.2016.12.016)
Supplement: Document S2. Article plus Supplemental Information [file mmc2.pdf]

Molecular Cell

Article

DNA Double-Strand Break Resection Occurs during Non-homologous End Joining in G1 but Is Distinct from Resection during Homologous Recombination

Graphical Abstract

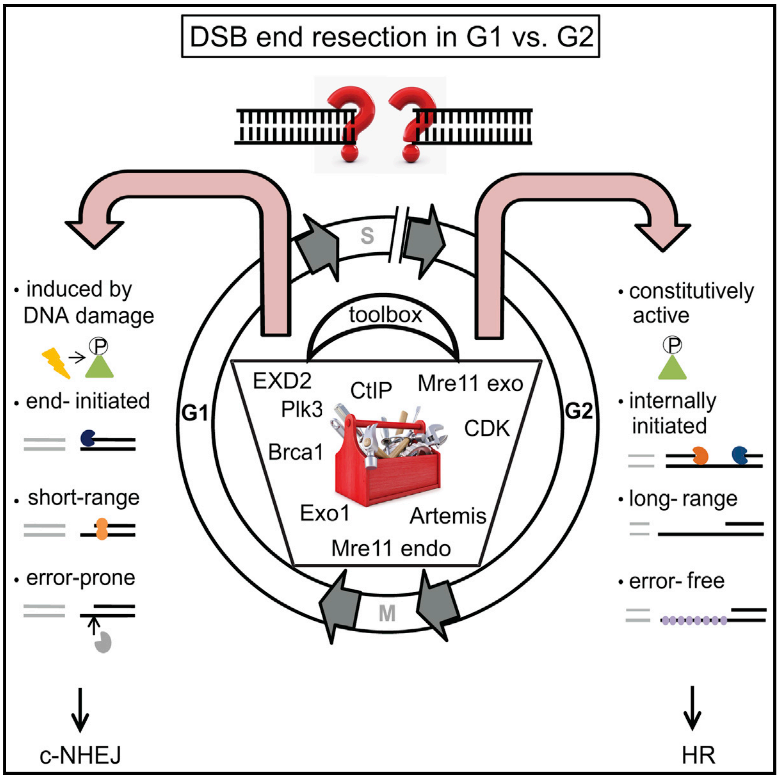

Authors

Ronja Biehs, Monika Steinlage, Olivia Barton, ..., Atsushi Shibata, Penny A. Jeggo, Markus Löbrich

Correspondence

shibata.at@gunma-u.ac.jp (A.S.),
p.a.jeggo@sussex.ac.uk (P.A.J.),
lobrich@bio.tu-darmstadt.de (M.L.)

In Brief

Biehs et al. identify and characterize a resection-dependent canonical non-homologous end joining process that operates in G1 phase cells. Resection in G2 leads to homologous recombination. Resection in G1 is distinct from that because the same toolbox of resection factors is orchestrated in a way that is compatible with an end-joining process.

Highlights

- DSBs in G1 are repaired by resection-independent or resection-dependent c-NHEJ
- Resection-dependent c-NHEJ is induced by Plk3-dependent CtIP interaction with Brca1
- G1 resection is executed from DSB ends by Mre11 exo, EXD2, and Exo1, but not Mre11 endo
- Artemis completes resection in G1, forming deletions and, potentially, translocations

Biehs et al., 2017, Molecular Cell *65*, 671–684
February 16, 2017 © 2016 The Author(s). Published by Elsevier Inc.
http://dx.doi.org/10.1016/j.molcel.2016.12.016

Molecular Cell
Article

CellPress

DNA Double-Strand Break Resection Occurs during Non-homologous End Joining in G1 but Is Distinct from Resection during Homologous Recombination

Ronja Biehs,[1,4] Monika Steinlage,[1,4] Olivia Barton,[1,4] Szilvia Juhász,[1] Julia Künzel,[1] Julian Spies,[1] Atsushi Shibata,[2,3,*] Penny A. Jeggo,[2,*] and Markus Löbrich[1,5,*]
[1]Radiation Biology and DNA Repair, Darmstadt University of Technology, 64287 Darmstadt, Germany
[2]Genome Damage and Stability Centre, University of Sussex, Brighton BN1 9RQ, UK
[3]Advanced Scientific Research Leaders Development Unit, Gunma University, Maebashi, Gunma 371-8511, Japan
[4]Co-first author
[5]Lead Contact
*Correspondence: shibata.at@gunma-u.ac.jp (A.S.), p.a.jeggo@sussex.ac.uk (P.A.J.), lobrich@bio.tu-darmstadt.de (M.L.)
http://dx.doi.org/10.1016/j.molcel.2016.12.016

SUMMARY

Canonical non-homologous end joining (c-NHEJ) repairs DNA double-strand breaks (DSBs) in G1 cells with biphasic kinetics. We show that DSBs repaired with slow kinetics, including those localizing to heterochromatic regions or harboring additional lesions at the DSB site, undergo resection prior to repair by c-NHEJ and not alt-NHEJ. Resection-dependent c-NHEJ represents an inducible process during which Plk3 phosphorylates CtIP, mediating its interaction with Brca1 and promoting the initiation of resection. Mre11 exonuclease, EXD2, and Exo1 execute resection, and Artemis endonuclease functions to complete the process. If resection does not commence, then repair can ensue by c-NHEJ, but when executed, Artemis is essential to complete resection-dependent c-NHEJ. Additionally, Mre11 endonuclease activity is dispensable for resection in G1. Thus, resection in G1 differs from the process in G2 that leads to homologous recombination. Resection-dependent c-NHEJ significantly contributes to the formation of deletions and translocations in G1, which represent important initiating events in carcinogenesis.

INTRODUCTION

DNA double-strand breaks (DSBs) are repaired by two major pathways: canonical non-homologous end joining (c-NHEJ) or homologous recombination (HR) (Jackson and Bartek, 2009; Lukas and Lukas, 2013). c-NHEJ rejoins DSBs using little or no sequence homology and functions throughout the cell cycle. Key players are the Ku70/80 heterodimer and the DNA-dependent protein kinase catalytic subunit (DNA-PKcs), which is recruited to DNA-bound Ku70/80, generating the DNA-PK holoenzyme (Jette and Lees-Miller, 2015). DNA ligase IV (Lig4), XRCC4, XRCC4-like factor (XLF)/Cernunnos, and parolog of XRCC4 and XLF (PAXX) operate during later stages of c-NHEJ (Ochi et al., 2015). HR is initiated by CtBP-interacting protein (CtIP)-dependent resection, creating 3′ single-stranded DNA (ssDNA) overhangs at DSB ends (Huertas and Jackson, 2009; Sartori et al., 2007). HR exerts its major role at stalled or collapsed replication forks in S phase but also contributes to DSB repair during G2 (Moynahan and Jasin, 2010).

DSB repair can occur by alternative NHEJ mechanisms, termed alt-NHEJ (Nussenzweig and Nussenzweig, 2007). alt-NHEJ involves CtIP-dependent resection, poly-(ADP-ribose)-polymerases (PARPs), Lig1 or 3 instead of Lig4, and XRCC1 (Lee-Theilen et al., 2011; Wang et al., 2005). CtIP-dependent end joining occurs in G1 cells (Yun and Hiom, 2009) and contributes to translocation formation at restriction enzyme- or ionizing radiation (IR)-induced DSBs (Zhang and Jasin, 2011; Barton et al., 2014). Polo-like kinase 3 (Plk3) phosphorylates CtIP in G1, promoting limited end resection and enhancing translocations (Barton et al., 2014). Thus, various lines of evidence demonstrate that end resection occurs in G1, although it is unclear whether the pathway(s) utilizing resected ends are restricted to alt-NHEJ or include a c-NHEJ process.

IR-induced DSBs are repaired with biphasic kinetics involving a fast and a slow process (DiBiase et al., 2000; Riballo et al., 2004). In G1, cells defective in c-NHEJ fail to repair DSBs by either process (Riballo et al., 2004). In G2, c-NHEJ deficiency affects only the fast process, whereas the slow process represents HR (Beucher et al., 2009). The slow processes in G1 and G2 repair heterochromatic DSBs (about 15%–20% of all DSBs) (Goodarzi et al., 2008; Riballo et al., 2004) and require the Artemis nuclease, suggesting involvement of end-processing steps (Riballo et al., 2004).

Loss of downstream HR factors (e.g., Brca1, Brca2, Rad51, and Rad54) diminishes HR, causing unrepaired DSBs in the slow component in G2 (Beucher et al., 2009; Shibata et al., 2011). Loss of CtIP also abolishes HR in G2 because resection is not initiated. However, it does not result in unrepaired DSBs because c-NHEJ, which is normally restricted to the fast component in G2, can repair unresected breaks (Shibata et al., 2011). Thus, a repair factor may function in the repair process even

CrossMark

This is an open access article under the CC BY license (http://creativecommons.org/licenses/by/4.0/).

though its loss does not confer a repair defect (e.g., CtIP in G2). This is important because CtIP depletion does not cause a repair defect in G1 but strongly diminishes chromosome translocations (Barton et al., 2014). Therefore, we considered the possibility that the slow process in G1 represents a resection-dependent process (as in G2) and that preventing resection by CtIP depletion causes a pathway switch from resection-dependent to resection-independent c-NHEJ, explaining the lack of a repair defect and reduced chromosome translocations (making the assumption that resected DSBs are more prone to undergo mis-rejoining than unresected DSBs).

We clarify this provocative possibility and verify that the slow repair component in G1 represents a resection-dependent rejoining process. The process utilizes c-NHEJ and not alt-NHEJ factors. Thus, the two main repair processes in G1 human cells are resection-independent and resection-dependent c-NHEJ. Resection-dependent c-NHEJ avidly forms IR-induced translocations, highlighting its physiological relevance. We then investigated the factors regulating resection-dependent c-NHEJ and identified striking differences to the resection process in G2. First, resection-dependent c-NHEJ is initiated after DSB induction by Plk3, which phosphorylates CtIP at Ser327 to mediate CtIP-Brca1 interaction. Thus, in contrast to G2, where cyclin-dependent kinases (CDKs) constitutively phosphorylate CtIP, the initiation of resection in G1 is inducible. Following initiation by Plk3/CtIP/Brca1, Mre11 exonuclease, EXD2, and Exo1 execute resection, and Artemis completes the process. Mre11 endonuclease activity is dispensable for resection in G1, suggesting that resection commences from the DSB end and not internally as in G2 (Shibata et al., 2014). Our findings reveal differences in resection between G1 and G2, enhancing our understanding of DSB repair in human cells and facilitating the design of approaches to reactivate HR in G1 for gene targeting (Orthwein et al., 2015).

RESULTS

Slow Artemis-Dependent c-NHEJ Promotes Translocation Formation in G1

We enumerated translocations forming in G1 human fibroblasts by premature chromosome condensation (PCC) combined with fluorescence in situ hybridization (FISH). We harvested asynchronous 82-6 fibroblasts at defined times after X-ray IR (X-IR) and fused them with mitotic HeLa cells to promote PCC of fibroblast chromosomes. G1 PCC spreads were distinct from G2 spreads and from mitotic HeLa cells by their one-chromatid morphology and the G1-specific marker CDT1 (Nishitani et al., 2001). PCC spreads from S phase cells displayed massive chromosome breakage and were also excluded from the analysis (Gotoh and Durante, 2006). Nocodazole was added to prevent G2 cells from entering G1, and control experiments confirmed that cells were irradiated and maintained in G1 (Figures S1 and S2A–S2C). We stained chromosomes 1, 2, and 4 by FISH and identified chromosome breaks as stained fragments and translocations by the appearance of chromosomes with color junctions (Figure 1A). Chromosome breaks were rejoined with biphasic kinetics (Figure 1B), consistent with DSB rejoining assessed by γH2AX analysis or pulsed field gel electrophoresis (Riballo et al., 2004). Some translocations formed within 6 hr after X-IR, when most chromosome breaks were repaired. However, between 6 and 14 hr after X-IR, translocations doubled, but few breaks were repaired, demonstrating that the slow process is particularly error-prone (Figure 1C; Barton et al., 2014).

To investigate the process causing translocations, we treated cells with a PARP inhibitor or small interfering RNA (siRNA) Lig1/3 (siLig1/3) but found no effect on chromosome break repair or translocation formation (Figures 1B and 1C), demonstrating that X-IR-induced translocations in G1 human cells do not form by alt-NHEJ. Next, we added a DNA-PK inhibitor 6 hr after X-IR (when the fast repair component is completed) and observed elevated chromosome breaks and diminished translocations 14 hr after X-IR compared with untreated cells (Figure 1D). Indeed, translocation levels after DNA-PK inhibitor addition at 6 hr are only slightly increased compared with translocations arising in the first 6 hr when DNA-PKcs was active. This suggests that a slow c-NHEJ process involving DNA-PKcs substantially contributes to X-IR-induced chromosome translocation formation.

Because Artemis is essential for slow DSB repair, we next carried out combined PCC/FISH analysis in Artemis-deficient fibroblasts. We observed elevated unrepaired breaks and diminished translocations 14 but not 6 hr after X-IR (Figure 1E). Thus, Artemis deficiency specifically affects the slow component of translocation formation, confirming that such translocations arise from the slow DSB repair process.

To confirm that the slow Artemis-dependent translocations arise from c-NHEJ, we employed a semi-automated microscopic approach that assesses repair kinetics by γH2AX focus analysis in defined cell-cycle phases (Figure S2D). First, we investigated DNA-PK involvement during the slow repair process in G1. Using 7 Gy, the same dose used for translocation measurements, we added the DNA-PK inhibitor 6, 8, 10, and 12 hr after X-IR and analyzed γH2AX foci at 14 hr in G1 cells. Inhibitor addition at all time points strongly impaired DSB repair, consistent with the notion that DNA-PK is bound to break ends throughout the slow repair process (Figure 1F). Furthermore, PARP inhibition did not affect γH2AX focus levels 14 hr after X-IR in control, XLF-deficient or Lig4-mutated fibroblasts (Figure 1G), although it increased focus numbers in HeLa cells treated with siKu80 (Figure S2E).

To confirm that DNA-PK and Artemis operate in the same slow repair process, we added the DNA-PK inhibitor to G1 phase Artemis-deficient and control fibroblasts. Using 2 Gy, we added the inhibitor 4 hr after X-IR (when the fast DSB repair process had completed) and scored γH2AX foci 8 and 10 hr after X-IR. Of note, DNA-PK inhibition did not affect the focus level of Artemis-deficient cells but increased focus numbers in control cells to that of Artemis-deficient cells, demonstrating that DNA-PK and Artemis function during slow DSB repair (Figure 1H). Collectively, we show that the slow Artemis-dependent component of translocation formation and DSB repair represents a c-NHEJ process and that human cells do not employ alt-NHEJ as long as Ku is present.

Artemis and CtIP Function during Slow DSB Repair in G1

The generation of X-IR-induced translocations in human cells by a c-NHEJ process rather than alt-NHEJ is consistent with results

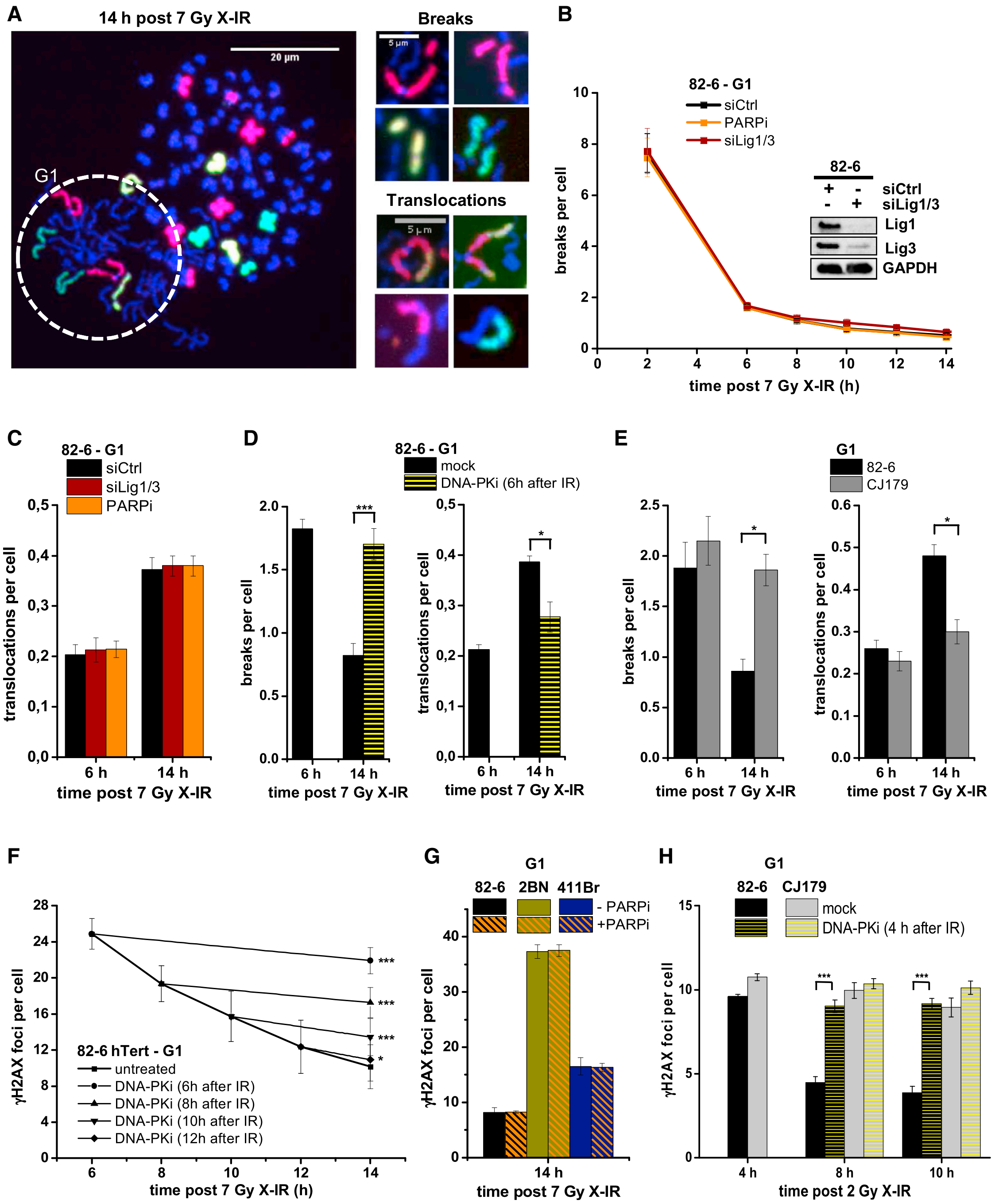

Figure 1. A Slow Artemis-Dependent c-NHEJ Process Promotes Translocation Formation in G1

(A) Left: FISH-stained G1 PCC spread from 82-6 control cells (dashed circle) fused with mitotic HeLa cells. Right: chromosome breaks and translocations in G1 PCC spreads.

(B and C) Chromosome breaks (B) and translocations (C) in G1 82-6 cells treated with siLig1/3 or PARP inhibitor (PARPi). Data are mean ± SEM.

(D) Chromosome breaks and translocations in G1 82-6 cells treated with DNA-PK inhibitor (DNA-PKi) 6 hr after X-IR. Data are mean ± SEM.

(E) Chromosome breaks and translocations in G1 82-6 and Artemis-deficient CJ179 cells. Data are mean ± SEM.

(F) γH2AX foci in G1 82-6 cells treated with DNA-PKi at various times after X-IR. Data are mean ± SEM.

(G) γH2AX foci in G1 82-6, XLF-deficient 2BN and Lig4-mutated 411Br cells treated with PARPi. Data are mean ± SEM.

(H) γH2AX foci in G1 82-6 and CJ179 cells treated with DNA-PKi 4 hr after X-IR. Data are mean ± SEM.

See also Figures S1 and S2.

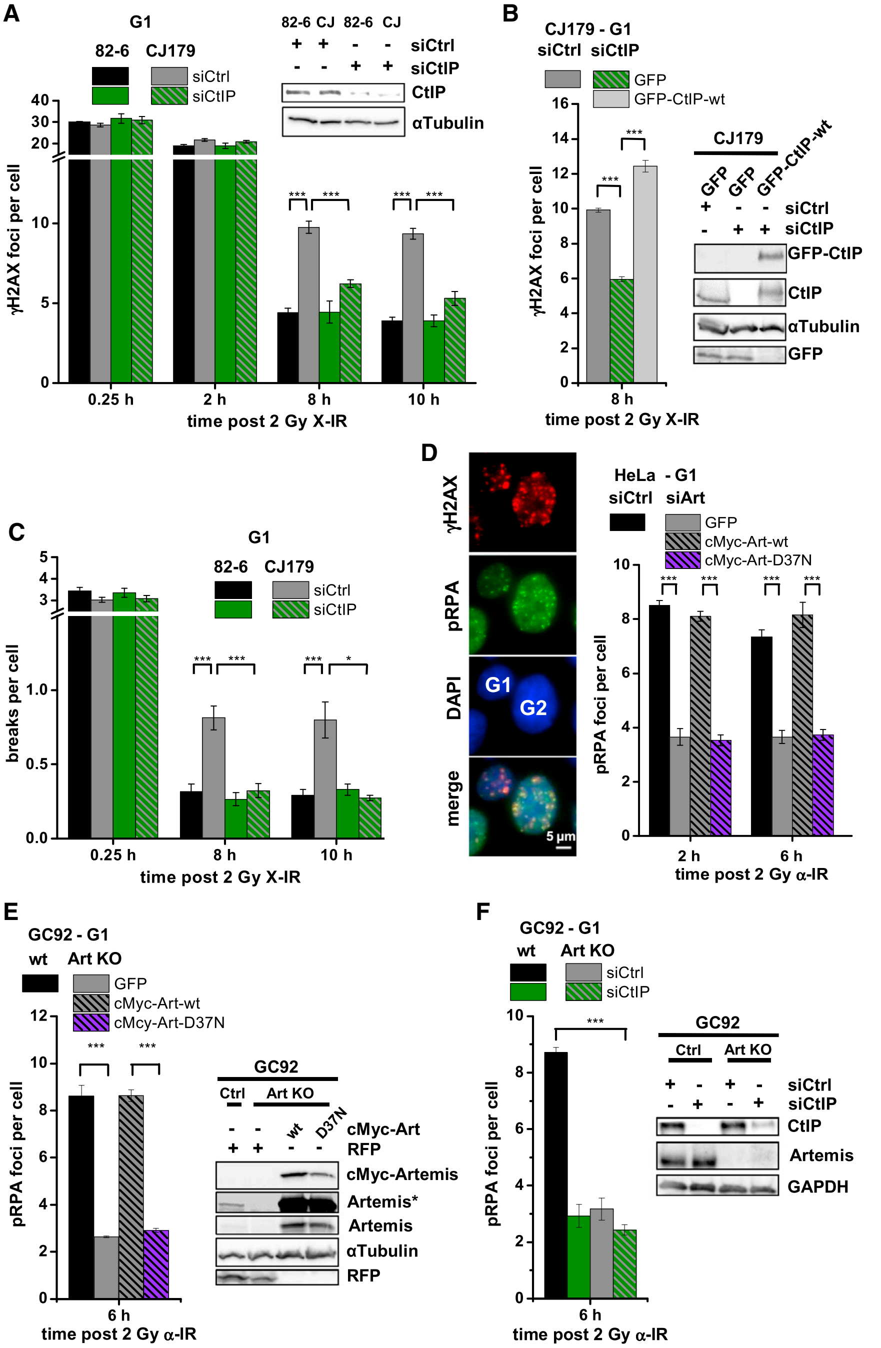

Figure 2. Artemis and CtIP Function during Slow DSB Repair and Promote Resection in G1

(A) γH2AX foci in G1 82-6 and CJ179 cells treated with siCtIP. Data are mean ± SEM.

(B) γH2AX foci in CJ179 cells treated with siCtIP. Cells were transfected with GFP or GFP-CtIP-WT constructs, and γH2AX foci were analyzed in GFP$^+$ G1 cells. Data are mean ± SEM.

(C) Chromosome breaks in G1 82-6 and CJ179 cells treated with siCtIP. Data are mean ± SEM.

(D) pRPA foci in G1 HeLa cells treated with siArtemis. Cells were transfected with GFP or cMyc-Artemis plasmids, and foci were analyzed in GFP/cMyc$^+$ G1 cells. Data are mean ± SEM.

(E) pRPA foci in G1 GC92 WT and CRISPR/Cas9-generated Artemis KO cells. Cells were transfected with GFP or cMyc-Artemis constructs, and pRPA foci were analyzed in GFP/cMyc$^+$ G1 cells. Data are mean ± SEM.

(F) pRPA foci in G1 GC92 WT and Artemis KO cells treated with siCtIP. Data are mean ± SEM.

See also Figure S3.

employing designer nucleases (Ghezraoui et al., 2014). We observed previously that CtIP contributes to the slow component of X-IR-induced translocations in G1 human cells, although the underlying repair pathway was not examined (Barton et al., 2014). This raised the possibility that the slow c-NHEJ process in G1 involves Artemis and CtIP. Therefore, we measured the kinetics of DSB repair in siCtIP-depleted G1 fibroblasts. In wild-type (WT) cells, we observed similar γH2AX focus numbers at all times, analyzed with or without siCtIP (Figure 2A; Barton et al., 2014). In G2, siCtIP abolishes resection and HR but does not cause a repair defect because c-NHEJ can be used if resection is not initiated. Consequently, co-depletion of CtIP/Brca1 or CtIP/Brca2 relieves the repair defect caused by loss of Brca1 or Brca2, respectively (Kakarougkas et al., 2013; Shibata et al., 2011).

Thus, we considered that siCtIP might similarly cause a switch from a resection-dependent to a resection-independent process in G1. Because Artemis is essential for the slow repair process in G1, as are Brca1 and Brca2 in G2, we examined whether siCtIP affects repair in Artemis mutants in G1. As expected, we observed the same level of γH2AX foci in Artemis-deficient and control fibroblasts at 15 min and 2 hr but higher levels in Artemis-deficient fibroblasts 8 and 10 hr after X-IR (Figure 2A). Strikingly, siCtIP rescued the repair defect of G1 Artemis mutants (Figure 2A), and overexpression of siRNA-resistant GFP-tagged CtIP in siCtIP-treated Artemis mutants restored the repair defect (Figure 2B). A similar rescue by siCtIP was observed in another cell system (Figure S3A). We conclude that Artemis and CtIP function during slow DSB repair in G1. The finding that Artemis but not CtIP deficiency confers a repair defect strongly suggests that Artemis functions downstream of CtIP, reflective of the situation in G2, where several factors (e.g., Brca1 and Brca2) function downstream of CtIP to promote HR and that their combined depletion with CtIP rescues their repair defects. This was confirmed by investigating chromosome breaks in G1 cells using PCC/FISH. We observed similar initial breakage levels for all

conditions, a pronounced repair defect in Artemis-deficient fibroblasts 8 and 10 hr after X-IR, and rescue by siCtIP (Figure 2C). In conclusion, Artemis and CtIP function during slow DSB repair, which confirms our translocation measurements and shows that this DSB repair process causes slow translocation formation.

Artemis and CtIP Promote Resection in G1

To gain direct evidence for resection during slow DSB repair in G1, we examined phospho-replication protein A (pRPA) foci by immunofluorescence using our semi-automated microscopic approach (Figure S2D). Because pRPA foci are difficult to detect after X-IR in G1, we exploited α particle IR (α-IR), which induces multiple damages in close proximity, creating complex DSBs. DSB end complexity impedes NHEJ and slows DSB repair, which promotes resection, pRPA focus formation, and HR usage in G2 (Barton et al., 2014; Shibata et al., 2011).

Indeed, pRPA foci are readily observed in G1 2 and 6 hr after α-IR and require CtIP (Barton et al., 2014). siArtemis reduced pRPA focus numbers (Figure 2D), demonstrating that Artemis and CtIP promote resection in G1. We transfected Artemis-depleted cells with siRNA-resistant cMyc-tagged Artemis constructs and enumerated pRPA foci in cMyc^{+} G1 cells. WT but not nuclease-deficient Artemis restored the resection defect conferred by siArtemis (Figure 2D). We also generated an Artemis knockout (KO) cell line by CRISPR/Cas9 technology and observed fewer pRPA foci in G1 Artemis KO cells than in control cells. The diminished focus level was restored by WT but not nuclease-defective Artemis (Figure 2E). The resection defect in Artemis KO cells was similar to that of siCtIP cells, and siCtIP in Artemis KO cells caused no further defect (Figure 2F). Finally, assessment of ssDNA in G1 cells by enumerating bromodeoxyuridine (BrdU) foci confirmed that Artemis is required for resection (Figure S3B). To confirm that resection after α-IR represents the same Artemis/CtIP-dependent slow c-NHEJ pathway uncovered in the Artemis rescue experiments, we examined γH2AX foci after α-IR in G1. We observed delayed repair kinetics compared with X-IR and a requirement for Artemis and CtIP (Figure S3C; Barton et al., 2014). Moreover, DNA-PK but not PARP inhibition conferred a repair defect (Figure S3C). Given the slow kinetics, Artemis requirement, and PARP independence, the repair of α-IR-induced DSBs appears to represent the same c-NHEJ pathway that repairs $\sim$15%–20% of DSBs after X-rays.

We also measured pRPA foci after 20-Gy X-rays, a dose giving similar focus numbers as 2-Gy α-IR. siArtemis and siCtIP reduced pRPA levels as for α-IR (Figure S3D). We then examined whether Ku was retained at resected DSBs by co-staining against pRPA and Ku80. Strikingly, although the samples showed significant Ku80 background staining, nearly every G1 pRPA focus co-localized with a Ku80 focus (Figure S3E). As a control, we co-stained Ku80 and Rad51 in G2 cells using the same conditions (20 Gy, 4 hr) plus a lower dose at a later time point (4 Gy, 8 hr). We rarely observed co-localization of Ku80 and Rad51, demonstrating antibody specificity (Figure S3F). Importantly, a recent paper showed that Ku is removed from resected DSBs in G2 concomitant with Rad51 loading (Chanut et al., 2016). We conclude that Artemis nuclease, together with CtIP, promotes DSB resection in G1, although the extent of resection is more limited than in G2 because detecting pRPA requires high doses or complex DSBs. We further propose that Ku remains bound during resection in G1.

Molecular Characterization of G1 Resection

To molecularly characterize the Artemis- and CtIP-dependent resection process, we employed a reporter assay containing two I-SceI restriction sites located 3.2 kilobase pairs (kbp) apart. Joining of the distant DSB ends causing loss of the intervening fragment was monitored (Figure 3A). The joining events arise in G1 (Barton et al., 2014) and require CtIP (Rass et al., 2009), suggesting that they necessitate some level of resection. Thus, we examined whether this assay selectively monitors the resection-dependent slow repair process. We first enumerated γH2AX foci that arise following I-SceI transfection. Control cells showed no significant focus induction over background, whereas siDNA-PKcs or usage of Artemis KO cells increased focus numbers (Figure 3B), suggesting that repair of these I-SceI-induced DSBs requires Artemis and DNA-PKcs. Notably, siCtIP had no effect in control cells but reduced the elevated focus numbers observed in Artemis KO cells (Figure 3B). This recapitulates our findings after X-IR, suggesting that this assay monitors the slow DSB repair process. Next, we used the Artemis KO cells containing the reporter and observed a complete reduction of end joining events involving loss of the 3.2-kbp fragment, which was restored by WT but not nuclease-deficient Artemis (Figure 3C). siCtIP reduced end joining events in control but not in Artemis KO cells (Figure 3D). Collectively, these findings show that the diminished end joining events in Artemis KO cells arise because of unrepaired DSBs, whereas siCtIP reduces end joining involving loss of the intervening fragment at the expense of events that escape detection in the assay (Figure 3A). Sequence analysis of the repair junctions in control cells revealed that loss of the intervening fragment is often associated with additional deletions (consistent with the notion that this assay monitors resection-dependent end joining) and frequently involves micro-homology usage (Figure 3E; Table S1).

We also investigated the role of alt-NHEJ and c-NHEJ in this reporter assay. Depletion of Ku increased the frequency of events (Figure 3F), consistent with the observation that alt-NHEJ can effect rejoining without Ku (Guirouilh-Barbat et al., 2004). In contrast, siDNA-PKcs and siLig4 substantially reduced end joining, whereas siLig1/3 had no significant effect on rejoining frequency, deletion size, or micro-homology usage (Figures 3E and 3F; Table S1). These data demonstrate that this reporter assay monitors a c-NHEJ process and that alt-NHEJ has no significant role in Ku-proficient cells. Thus, the reporter assay confirms the results obtained from the analysis of DSB repair pathway usage after IR.

Similar and Distinct Nuclease Requirements for Resection in G1 versus G2

Having established that Artemis and CtIP promote a resection-dependent slow NHEJ process in G1, we examined whether resection proceeds similar to that in G2. First, we asked which additional nucleases execute resection in G1 and investigated

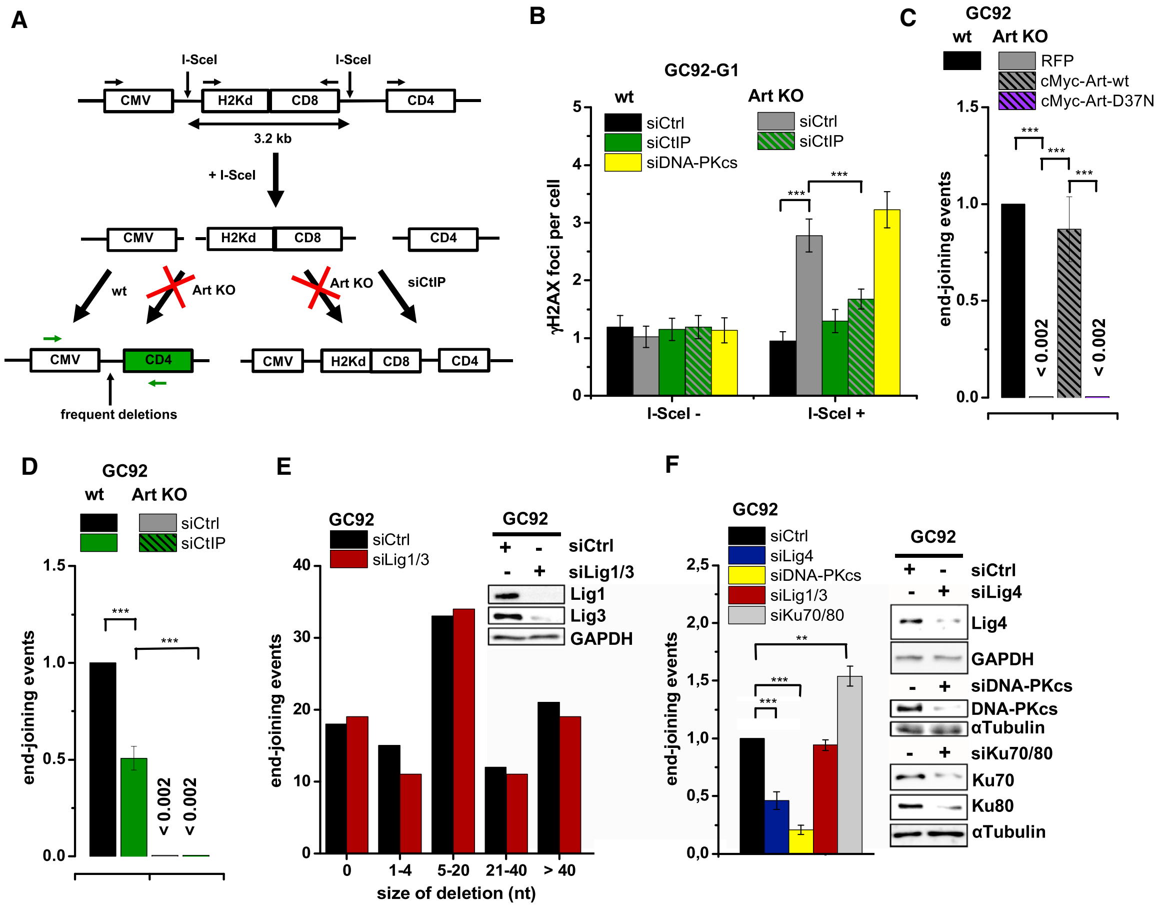

Figure 3. Molecular Characterization of G1 Resection

(A) Schematic of the NHEJ reporter assay. The repair of two I-SceI-induced DSBs can result in loss of the intervening fragment, which is detected by a CD4$^+$ signal (Rass et al., 2009). CD4$^+$ clones were amplified by PCR (green arrows) across the repair site and sequenced. Repair of the two DSBs can also occur without loss of the intervening fragment, which escapes detection.

(B) γH2AX foci in GC92 WT and Artemis KO cells treated with siDNA-PKcs or siCtIP. Cells were transfected with I-SceI, and foci were scored in I-SceI$^+$ and I-SceI$^-$ cells (identified by immunofluorescence [IF] against I-SceI). Data are mean ± SEM.

(C) End joining events in GC92 WT and Artemis KO cells containing the NHEJ reporter substrate. Cells were transfected with RFP or cMyc-Artemis constructs. Events were quantified by the fraction of CD4$^+$ and RFP/cMyc$^+$cells relative to all RFP/cMyc$^+$cells, and results were normalized to WT cells. Data are mean ± SEM.

(D) End joining events in GC92 WT and Artemis KO cells treated with siCtIP. Data are mean ± SEM.

(E) Distribution of deletion sizes obtained from the sequence analysis of GC92 WT and siLig1/3-treated cells. nt, nucleotide.

(F) End joining events in GC92 cells treated with siKu70/80, siLig4, siLig1/3, or siDNA-PKcs. Data are mean ± SEM.

See also Table S1.

Mre11, EXD2, Exo1, and Bloom syndrome mutated protein (BLM)/DNA2. We applied the three approaches described in Figures 2 and 3 to monitor resection-dependent slow NHEJ, assessing rescue of the Artemis repair defect in G1, pRPA focus formation in G1, and G1-specific end joining events in the reporter assay. Because Mre11 is required to activate ataxia telangiectasia mutated (ATM), its loss causes a repair defect in this process (Riballo et al., 2004), precluding analysis by siRNA. We therefore inhibited Mre11 nuclease activities by small-molecule inhibitors that selectively target its endo- or exonuclease activities (Shibata et al., 2014) without affecting ATM activation (Figure S4A). Inhibition of Mre11's endonuclease activity did not affect the γH2AX focus or chromosome break level of Artemis-deficient cells (Figure 4A; Figure S4B), pRPA levels (Figure 4B; Figure S3D), and the frequency of end joining in the reporter assay (Figure 4C). In contrast, inhibition of Mre11's exonuclease activity partially rescued the repair defect of Artemis-deficient cells (Figure 4A; Figure S4B), diminished the pRPA focus level (Figure 4B; Figure S3D), and reduced the frequency of end joining in the reporter assay (Figure 4C). These data suggest that Mre11 functions during resection in G1 as an exonuclease, whereas its endonuclease activity is dispensable. This differs from G2, where Mre11 endonuclease inhibition abolishes HR in a reporter assay (Figure S4C) and rescues the repair defect of Artemis-deficient cells (Figure S4D).

Examining Exo1's function, we obtained results nearly identical to Mre11 exonuclease inhibition. Specifically, siExo1 partially rescued the repair defect of Artemis-deficient cells

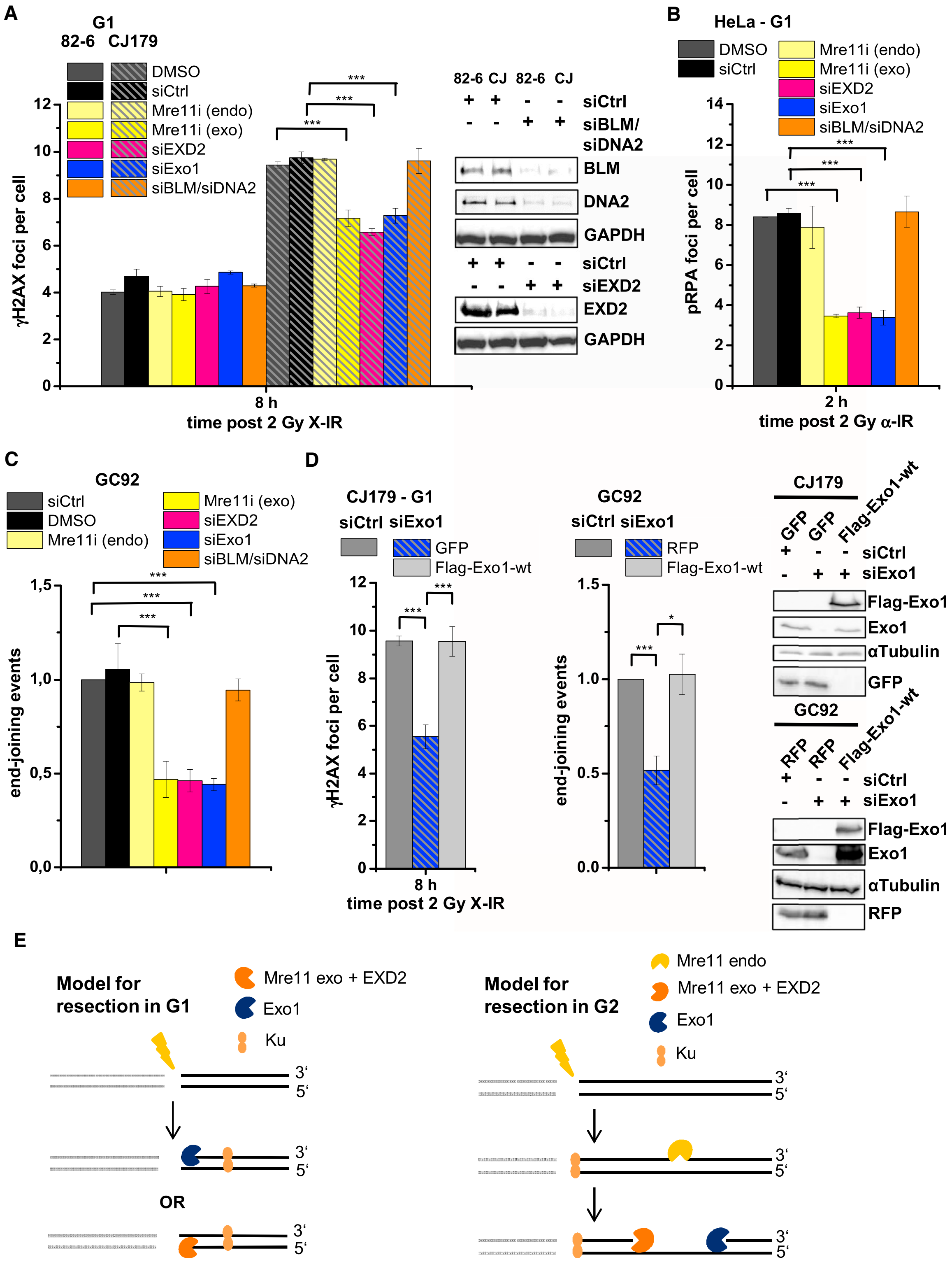

Figure 4. Similar and Distinct Nuclease Requirements for Resection in G1 versus G2

(A–C) γH2AX foci in G1 82-6 and CJ179 cells (A), pRPA foci in G1 HeLa cells (B), and end-joining events in GC92 cells (C). Cells were treated with an Mre11 endo- or exonuclease inhibitor, siEXD2, siExo1, or siBLM/siDNA2. Data are mean ± SEM.

(legend continued on next page)

(Figure 4A; Figure S4B), diminished the pRPA focus level (Figure 4B; Figure S3D), and reduced end joining in the reporter assay (Figure 4C). Expression of siRNA-resistant FLAG-tagged Exo1 restored the defect in Artemis-deficient cells treated with siExo1 and end joining in siExo1-treated cells in the reporter assay (Figure 4D). Additionally, siEXD2 had the same effect in our three assays as siExo1 or Mre11 exonuclease inhibition, whereas combined depletion of BLM and DNA2 was without effect (Figures 4A–4C). Collectively, these data show that the 5′-3′ exonuclease activity of Exo1 (Lee and Wilson, 1999) and the 3′-5′ exonuclease activities of Mre11 (Paull and Gellert, 1998) and EXD2 (Broderick et al., 2016) promote limited resection at slowly repairing DSBs in G1. Given that DNA-PKcs inhibition blocks the slow repair process and that Ku is required for DNA-PKcs binding, we propose that Ku70/80 remains bound to DSBs during resection, moving away from the break ends to expose DNA ends for nuclease access while limiting the extent of resected DNA (Figure 4E). This model is consistent with our analysis of Ku foci in G1 (Figure S3E). This suggests that resection in G1 is distinct from G2, where Mre11's endonuclease activity is proposed to initiate resection internal to the break end, followed by resection toward and away from the end by Mre11 exonuclease and Exo1, respectively (Figure 4E; Shibata et al., 2014). Consistent with this model, loss of Mre11 exonuclease activity or Exo1 causes a repair defect in G2 because the incompletely resected DSBs cannot be repaired by HR or NHEJ (Figure S4D).

Brca1 and 53BP1 Together Promote Resection-Dependent Slow DSB Repair in G1

Because Brca1 promotes resection in G2, we investigated whether it is also required for resection in G1 by applying our three assays. Using human fibroblasts, we observed that siBrca1 did not cause a repair defect in control cells but substantially rescued the defect of Artemis-deficient cells (Figure 5A; Figure S4B). We also observed diminished pRPA foci after siBrca1 (Figure 5B; Figure S3D) and reduced end joining in the reporter assay (Figure 5C). Expression of siRNA-resistant FLAG-tagged Brca1 restored the repair defect in siBrca1-treated Artemis-deficient cells and end joining in siBrca1-treated cells in the reporter assay (Figure 5D). Because loss of 53BP1 relieves the repair defect of Brca1 mutants in G2, we asked whether si53BP1 affects resection following siBrca1. Of note, combined si53BP1 and siBrca1 treatment increased pRPA focus numbers (Figure 5B) and end joining in the reporter assay (Figure 5C) to the level conferred by si53BP1 alone. This shows that Brca1 functions during resection-dependent c-NHEJ by counteracting 53BP1, similar to its described function during HR. Interestingly, si53BP1 led to increased pRPA foci numbers (Figure 5B) and elevated end joining events in the reporter assay (Figure 5C) compared with control cells, which were reduced to control levels after expression of siRNA-resistant HA-tagged 53BP1 (Figure S5A). This suggests that resection in the absence of 53BP1 is less restricted than in control cells. Significantly, depletion of Lig1/3 in si53BP1-treated cells nearly completely abolished end joining events in the reporter assay (Figure S5B), arguing that the repair process in 53BP1-defective cells differs from the resection-dependent c-NHEJ pathway described here. This is reminiscent of the situation in G2, where loss of 53BP1 channels DSB repair from gene conversion to single-strand annealing (Ochs et al., 2016). Thus, 53BP1 promotes resection-dependent c-NHEJ by regulating the extent of resection.

We next examined mouse embryonic fibroblasts (MEFs) carrying either Brca1-WT or Brca1-ΔBRCT, which lacks the interaction site with CtIP (Kakarougkas et al., 2013). siArtemis caused a repair defect in Brca1-WT but not Brca1-ΔBRCT MEFs (Figure 5E), which is rescued by siRNA-resistant cMyc-tagged Artemis (Figure S5C). We also observed diminished pRPA foci in Brca1-ΔBRCT compared with Brca1-WT MEFs (Figure 5F). To consolidate these functional studies, we measured Brca1 focus formation. We confirmed that Brca1 accumulation at DSBs after X-IR is visible but weaker in G1 compared with G2 (Figure S5D; Chapman et al., 2013; Escribano-Díaz et al., 2013; Feng et al., 2013). However, both G1 and G2 cells showed robust Brca1 accumulation at α-IR-induced DSBs (Figure S5E). Collectively, this suggests that Brca1 functions during resection-dependent slow DSB repair in G1 in a manner requiring its BRCA1 C-terminal (BRCT) domain. Furthermore, loss of Brca1 in G1 (unlike in G2) does not cause a DSB repair defect, demonstrating that it determines pathway choice in G1 but functions downstream of that step in G2.

Plk3 Promotes Resection-Dependent Slow DSB Repair in G1

Because Plk3 regulates CtIP in G1 (Barton et al., 2014), we investigated its role during slow repair in G1. We observed that, like siCtIP, siPlk3 does not cause a DSB repair defect but rescues the defect of siArtemis-treated HeLa cells (Figure 6A). Expression of siRNA-resistant FLAG-tagged Plk3 restored the repair defect in Artemis/Plk3-depleted cells (Figure 6B). siPlk3 in G2, where Plk3 is dispensable for CtIP regulation, did not rescue the defect of siArtemis-treated cells (Figure S6A). We confirmed these results with fibroblasts using siPlk3 and a Plk3 inhibitor (Plki) (Lansing et al., 2007; Figure S6B). Because Plk3 phosphorylates CtIP in G1 at Ser327 (Barton et al., 2014), we asked whether this phosphorylation event is required during resection-dependent repair. We co-depleted Artemis and CtIP in HeLa cells, transfected them with siRNA-resistant GFP-CtIP constructs, and enumerated γH2AX foci in G1. Notably, GFP-CtIP-WT and a phospho-mimic substitution at Ser327 (GFP-CtIP-S327E), but not a non-phosphorylatable mutant (GFP-CtIP-S327A), restored the Artemis repair defect in Artemis/CtIP-depleted cells (Figure 6C). The same result was obtained with fibroblasts (Figure S6C), confirming that CtIP phosphorylation at Ser327 is necessary for resection-dependent c-NHEJ.

(D) γH2AX foci in G1 CJ179 and end joining events in GC92 cells. Cells were treated with siExo1 and transfected with GFP, RFP, or FLAG-Exo1-WT constructs, and GFP^+, RFP^+ or $FLAG^+$ cells were analyzed. Data are mean ± SEM.
(E) Model for DSB end resection in G1 and G2. DNA-PKcs binding to Ku was omitted for clarity.
See also Figure S4.

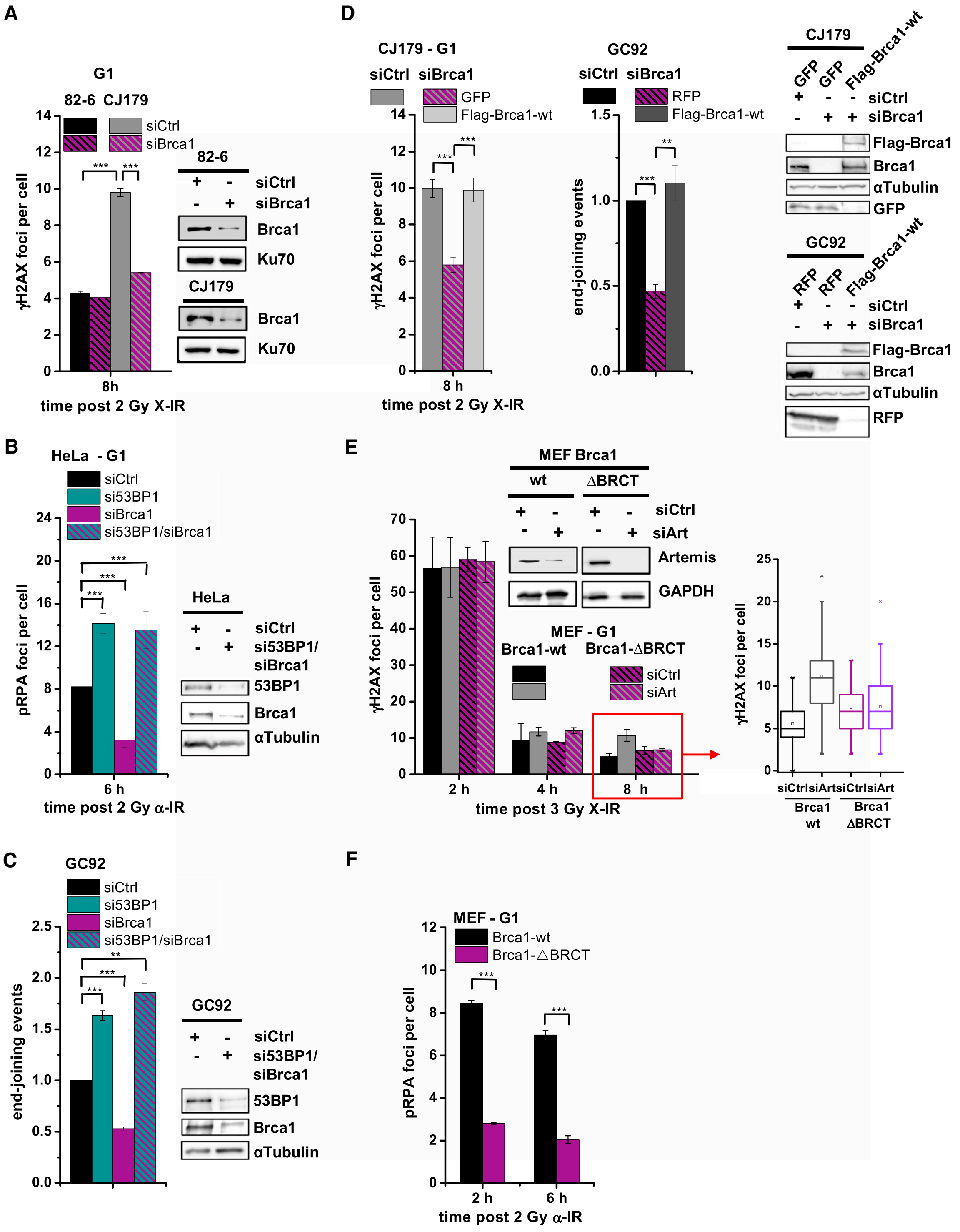

Figure 5. Brca1 and 53BP1 Together Promote Resection-Dependent Slow DSB Repair in G1

(A) γH2AX foci in G1 82-6 and CJ179 cells treated with siBrca1. Data are mean ± SEM.

(B) pRPA foci in G1 HeLa cells treated with si53BP1 and/or siBrca1. Data are mean ± SEM.

(C) End joining events in GC92 cells treated with si53BP1 and/or siBrca1. Data are mean ± SEM.

(legend continued on next page)

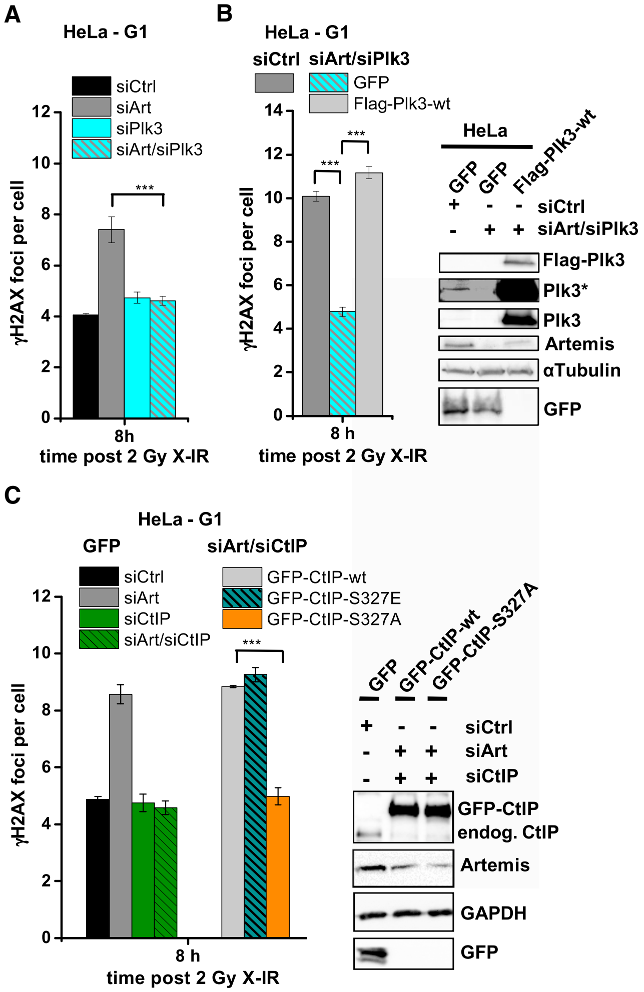

Figure 6. Plk3 Is Required for Resection-Dependent Slow DSB Repair in G1

(A) γH2AX foci in G1 HeLa cells treated with siArtemis and/or siPlk3. Data are mean ± SEM.

(B) γH2AX foci in G1 HeLa cells treated with siArtemis/siPlk3 and transfected with GFP or FLAG-Plk3-WT constructs, and GFP+ or FLAG+ G1 cells were analyzed. Data are mean ± SEM.

(C) γH2AX foci in G1 HeLa cells treated with siArtemis and/or siCtIP. Cells were transfected with GFP or GFP-CtIP constructs, and GFP+ G1-phase cells were analyzed. Data are mean ± SEM.

See also Figure S6.

CtIP Phosphorylation at Ser327 by Plk3 Mediates Interaction with Brca1 in G1

CtIP phosphorylation at Ser327 by CDKs in G2 mediates its interaction with Brca1 (Yu and Chen, 2004). Although this phosphorylation occurs constitutively in undamaged G2 cells, CtIP phosphorylation by Plk3 in G1 is only observed after IR (Barton et al., 2014). Because CtIP phosphorylation at Ser327 and Brca1's BRCT domain, which encompasses the CtIP interaction site, are required for slow repair in G1, we examined whether Brca1 and CtIP physically interact in G1 using co-immunoprecipitation analysis in synchronized G1 HeLa cells (Figure S7A). We confirmed that CtIP phosphorylation at Ser327 is only observed after IR and that Plki abolished the IR-induced signal (Figure 7A; Barton et al., 2014). Notably, Brca1 co-immunoprecipitated with CtIP closely followed the CtIP phosphorylation signal at Ser327; that is, it was absent in unirradiated samples, appeared 1 hr after IR, and was absent when the samples were treated with Plki (Figure 7A). In the reverse experiment, we observed strong levels of CtIP co-immunoprecipitated with Brca1 1 hr after IR only in non-Plki treated samples (Figure 7A). This analysis shows that CtIP and Brca1 physically interact in G1 in a damage-inducible manner that requires Plk3.

We next examined whether the damage-inducible CtIP-Brca1 interaction in G1 depends on CtIP phosphorylation at Ser327. We transfected HeLa cells with GFP-CtIP-WT or non-phosphorylatable GFP-CtIP-S327A, irradiated them or not, and immunoprecipitated GFP. The transfection stress caused >90% of the GFP-positive HeLa cells to arrest in G1 (Figure S7B). In cells transfected with GFP-CtIP-WT, we observed pronounced CtIP phosphorylation at Ser327 1 hr after IR but not without IR (Figure 7B). Brca1 did not co-immunoprecipitate with GFP-CtIP in unirradiated cells, but a robust signal was observed 1 hr after IR. Cells transfected with GFP-CtIP-S327A showed no CtIP phosphorylation at Ser327 and no detectable Brca1 (Figure 7B). Conversely, we detected a strong signal for GFP-CtIP co-immunoprecipitated with Brca1 in the irradiated GFP-CtIP-WT but not the GFP-CtIP-S327A sample (Figure 7B). These data show that CtIP and Brca1 physically interact in G1 in a damage-inducible manner dependent on CtIP phosphorylation at Ser327.

DISCUSSION

DSB resection can arise in G1 as well as S and G2, and Rad51 binding to extended ssDNA regions can occur in genetically manipulated G1 cells (Orthwein et al., 2015). However, Rad51 loading to ssDNA does not normally occur in G1, and resection is too limited to detect RPA binding microscopically, limiting our ability to study resection in G1.

Our study was initiated by the finding that CtIP depletion rescues the repair defect of Artemis mutants. Artemis is required for the slow DSB repair process that, after X-IR, repairs DSBs localizing to heterochromatic DNA regions. This has provided a readout to probe the role of additional factors for G1 resection. We reasoned that resection occurs in heterochromatin because repair is delayed (Goodarzi et al., 2008). Additionally, to study resection in G1, we utilized α-IR, which induces complex DSBs that are repaired with slow kinetics and undergo resection in G2 (Shibata et al., 2011). In G1, DSBs induced by α-IR give

(D) γH2AX foci in G1 CJ179 and end joining events in GC92 cells. Cells were treated with siBrca1 and transfected with GFP, RFP or FLAG-Brca1-WT constructs, and GFP+, RFP+ or FLAG+ cells were analyzed. Data are mean ± SEM.

(E) γH2AX foci in G1 Brca1-WT and Brca1-ΔBRCT MEFs treated with siArtemis. Data are mean ± SEM.

(F) pRPA foci in G1 Brca1-WT and Brca1-ΔBRCT MEFs. Data are mean ± SEM.

See also Figure S5.

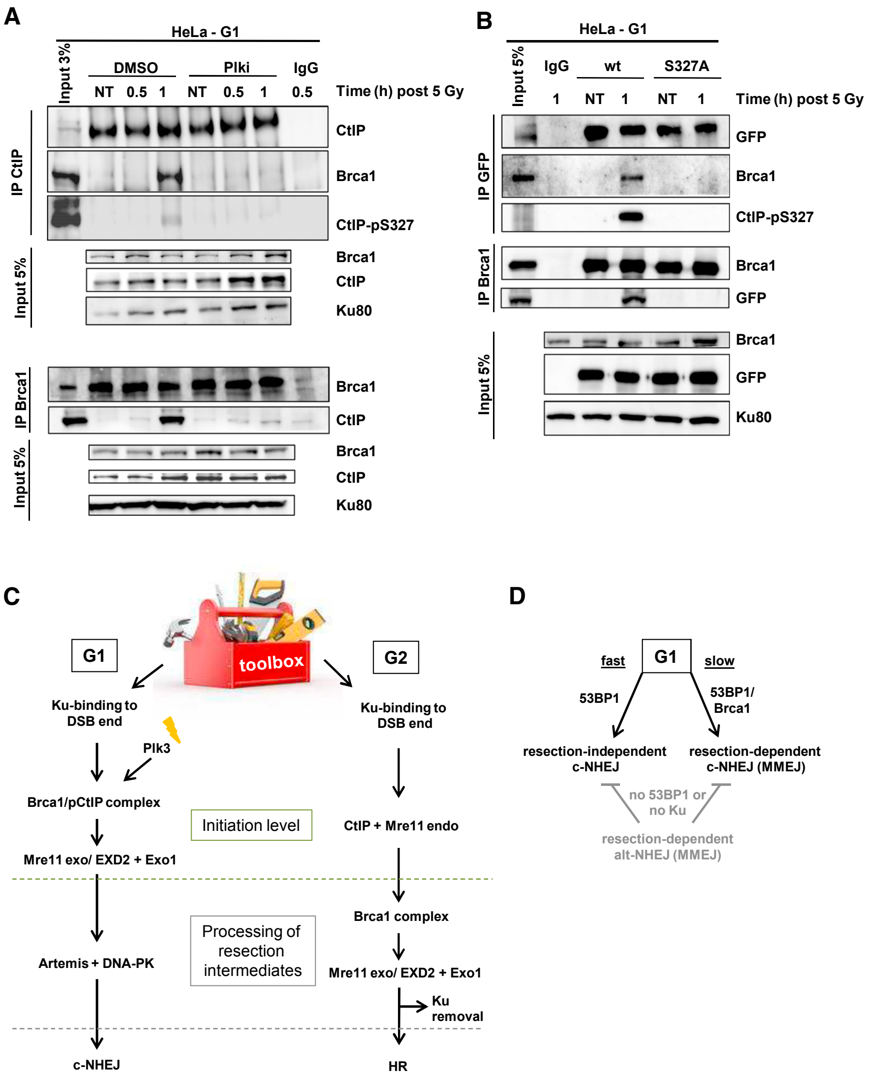

Figure 7. CtIP Phosphorylation at Ser327 by Plk3 Mediates Interaction with Brca1 in G1

(A) Interaction of CtIP and Brca1 in synchronized G1 HeLa cells treated with Plki.

(B) Interaction of CtIP and Brca1 in G1 HeLa cells transfected with GFP-CtIP-WT or GFP-CtIP-S327A. Brca1 or GFP/CtIP was immunoprecipitated from cell extracts, and protein levels were analyzed.

(C) Model summarizing the hierarchy of investigated factors involved in resection-dependent c-NHEJ in G1 in comparison with HR in G2.

(D) Model for DSB repair pathway choice in G1 human cells.

See also Figure S7.

rise to CtIP-dependent pRPA foci (Barton et al., 2014). This second approach fully consolidated the findings obtained with the Artemis rescue experiments.

As a third approach to study resection in G1, we used a reporter assay containing two I-SceI restriction sites that monitors end joining of the two distant ends with loss of the intervening fragment (Rass et al., 2009). We considered that such events represent slow DSB repair, whereas fast repair may promote end joining events without loss of the intervening fragment that escape detection in the assay. Because slow DSB repair involves resection, we reasoned that this assay might specifically monitor resection-dependent end joining. Thus, we exploited three independent methods to study resection-dependent slow DSB repair in G1. The reporter assay additionally revealed that resection-dependent end joining is associated with nucleotide losses of 5–20 bp, although the extent of resection might be larger for α-IR-induced DSBs.

Using these three approaches, we characterized the resection process in G1, revealing differences from G2 resection (Figure 7C). First, we showed that (as in G2 phase) the slow component of DSB repair in G1 represents a resection-dependent repair process. This is significant because previous work has established that slow DSB repair involves the c-NHEJ factors Lig4, XRCC4, and XLF (Beucher et al., 2009; Riballo et al., 2004). Here we show that DNA-PKcs inhibition at later times (when the fast repair process is completed) stops repair in G1, suggesting that DNA-PK is required for the slow process. This is distinct from the situation in G2, where DNA-PK is removed during resection. Thus, the slow component of DSB repair involves resection in G1 and G2, but, in G1, repair occurs via c-NHEJ, whereas, in G2, HR effects repair.

A second difference between resection in G1 versus G2 likely explains how DNA-PK binding to resected DSBs is maintained in G1 but prevented in G2. Although Mre11 initiates resection in G2 as an endonuclease internal to the DSB, this function of Mre11 is dispensable for resection in G1. Thus, it is likely that resection in G1 initiates from the DSB end by the exonuclease functions of Mre11, EXD2, and Exo1, which, because of their different polarity, can resect one or the other DNA strand. An interesting model is that Ku remains bound to the DSBs but moves away from the ends to allow nuclease access (i.e., translocates inward, a feature well described in biochemical studies; Turchi et al., 2000). In G2, in contrast, nucleolytic incision on the 5′ strand internal to the DSB is followed by resection toward and away from the DSB by the exonuclease functions of Mre11/EXD2 and Exo1, respectively. After removal of DNA-PK by still unknown processes, the large region of ssDNA likely prevents DNA-PK re-binding. Notably, maintained DNA-PK binding to DSBs during resection in G1 but not G2 (Figure 1F; Figure S3E) could also explain the more limited resection in G1.

The third difference between resection in G1 and G2 concerns the initiation step. In G2, CtIP is constitutively phosphorylated by

CDKs at Ser327, mediating interaction with Brca1 and promoting HR (Yu et al., 2006; Yun and Hiom, 2009), although the latter notion has been challenged (Reczek et al., 2013). Brca1 counteracts the anti-resection functions of 53BP1 and Rif1 (Chapman et al., 2013; Escribano-Díaz et al., 2013; Feng et al., 2013). In G1, IR activates Plk3, which phosphorylates CtIP at Ser327 (Barton et al., 2014). Hence, CtIP interacts with Brca1 in G1 only after damage induction. Because the CtIP interaction domain of Brca1 is required for resection, Brca1's role in promoting resection may be transiently kept in check to allow resection-independent c-NHEJ before activating the more error-prone resection-dependent c-NHEJ process.

Our findings also reveal distinctions in the commitment step to resection-dependent repair between G1 and G2 (Figure 7C). Depletion of Brca1 (and also Exo1 and Mre11 exonuclease) causes a repair defect in G2 because Brca1 lies downstream of CtIP-dependent initiation of resection (Kakarougkas et al., 2013). In contrast, Brca1 depletion does not cause a defect in G1, suggesting that it is required for the initiation process, which, if prevented, allows rejoining without resection. This might also explain the controversy concerning Brca1's role in G1 (because the assays used may or may not be specific for the described resection-dependent process) (Wu et al., 2010). Interestingly, our results show that Brca1 relieves a 53BP1 barrier to resection, defining a hitherto undescribed role for Brca1 in G1. Significantly, although 53BP1 creates a block to all resection and its loss allows unregulated resection and alt-NHEJ, the interplay between BRCA1 and 53BP1 promotes resection-dependent c-NHEJ (Figure 7D).

An important distinction between factors that initiate resection versus Artemis is that X-ray-induced DSBs are repaired without the initiating factors but remain unrepaired without Artemis. Thus, we propose that Artemis does not process the primary IR-induced DSBs as hypothesized previously (Riballo et al., 2004) but, rather, resolves intermediate structures that arise following resection by Exo1/EXD2 or Mre11 exonuclease, respectively. An interesting (although not the only) model is that 5′ or 3′ ssDNA overhangs are captured by a channel in DNA-PKcs, identified by structural studies, that is of the required size to allow passage of ssDNA but not double-stranded DNA (dsDNA) (Leuther et al., 1999; Williams et al., 2008, 2014). This could create a hairpin-like end necessitating Artemis for cleavage (Figure S7C). Our observation that Artemis is required for pRPA focus formation suggests that RPA binding only occurs after such cleavage. This model is appealing because it explains the absolute requirement for Artemis in removing trapped resection intermediates and reflects its role in cleaving hairpin intermediates during V(D)J recombination (Ma et al., 2002). The model is consistent with biochemical studies and explains why Ku and DNA-PKcs are required for efficient Artemis activity (Chang et al., 2015). Moreover, Artemis's endonucleolytic function downstream of initiation is consistent with the observed loss of nucleotides during resection-dependent c-NHEJ.

Micro-homology mediated end-joining (MMEJ) is a DSB rejoining process defined by short micro-homology usage (McVey and Lee, 2008). MMEJ is often taken to be synonymous with alt-NHEJ. However, the rejoining step of MMEJ could occur by c-NHEJ or alt-NHEJ. Our findings suggest that resection-dependent c-NHEJ represents MMEJ because alt-NHEJ does not substantially contribute to DSB rejoining or translocation formation in G1 human cells, functioning only in the absence of Ku or 53BP1 (Figure 7D). Alt-NHEJ has a greater function in rodent cells, where it contributes to translocations, potentially because of lower DNA-PK levels (Ghezraoui et al., 2014). Importantly, resection-dependent c-NHEJ significantly contributes to IR-induced translocations in human cells, consistent with the contribution of CtIP after DSB induction by restriction enzymes (Zhang and Jasin, 2011). Thus, our finding that CtIP (which was hitherto believed to promote alt-NHEJ) functions during c-NHEJ unifies these apparently contradicting notions. Thus, we propose that the slow component of DSB repair in G0/G1 phase human cells can result in MMEJ, with rejoining involving c-NHEJ and not alt-NHEJ.

In summary, we have identified and characterized a resection-dependent c-NHEJ process and revealed distinctions from the resection process during HR. Resection is activated in G1 by Plk3 which phosphorylates CtIP at Ser327, mediating its binding to Brca1, and is then executed by Exo1, EXD2, and Mre11 exonuclease. Mre11's endonuclease function, which initiates resection during HR in G2, is not involved. Finally, Artemis functions as an endonuclease downstream of the executing exonucleases to complete the process. DNA-PK coordinates the completion of repair by c-NHEJ. Thus, resection-dependent c-NHEJ uses the same toolbox of resection factors involved in HR but orchestrates them to be compatible with an end-joining process (Figure 7C).

STAR★METHODS

Detailed methods are provided in the online version of this paper and include the following:

- KEY RESOURCES TABLE
- CONTACT FOR REAGENT AND RESOURCE SHARING
- EXPERIMENTAL MODEL AND SUBJECT DETAILS
  - Cell Lines and Cell Culture
  - Bacterial Strains
- METHOD DETAILS
  - Generation of Artemis KO Cells with CRISPR/Cas9
  - RNA Interference and Plasmid Transfection
  - IR and Chemical Treatment
  - Chromosomal Analysis
  - Protein Extracts, Immunoprecipitation, and Immunoblotting
  - Immunofluorescence
  - Reporter Assays
  - Sequence Analysis in NHEJ Reporter Assay
  - Anti-pSer327 Antibody Preparation
  - Cell Synchronization and Flow Cytometry
- QUANTIFICATION AND STATISTICAL ANALYSIS

SUPPLEMENTAL INFORMATION

Supplemental Information includes seven figures and two tables and can be found with this article online at http://dx.doi.org/10.1016/j.molcel.2016.12.016.

AUTHOR CONTRIBUTIONS

R.B., M.S., and O.B. performed most experiments and interpreted the data. S.J., J.K., J.S., and A.S. participated in the experiments. A.S., P.A.J., and M.L. conceived the experiments. M.L. wrote the paper with help from P.A.J.

ACKNOWLEDGMENTS

We thank Tanya Paull for providing phospho-specific antibodies; Susan Lees-Miller for helpful discussions; and Junjie Chen, Zihua Gong, Hemmo Meyer, and Markus Wolf for experimental input. We thank Ratna Weimer, Bettina Basso, Christel Braun, and Cornelia Schmitt for technical assistance and Amira Elbakry for help with manuscript editing. Work in the M.L. laboratory is supported by the Deutsche Forschungsgemeinschaft (GRK1657), the European Union (grant RISK-IR), and the Bundesministerium für Bildung und Forschung (02NUK034A/037C and 02NUK016D/017E). P.A.J. was supported by the Medical Research Council (G000050 and G0500897).

Received: March 13, 2016
Revised: September 30, 2016
Accepted: December 19, 2016
Published: January 26, 2017

REFERENCES

Barton, O., Naumann, S.C., Diemer-Biehs, R., Künzel, J., Steinlage, M., Conrad, S., Makharashvili, N., Wang, J., Feng, L., Lopez, B.S., et al. (2014). Polo-like kinase 3 regulates CtIP during DNA double-strand break repair in G1. J. Cell Biol. *206*, 877–894.

Beucher, A., Birraux, J., Tchouandong, L., Barton, O., Shibata, A., Conrad, S., Goodarzi, A.A., Krempler, A., Jeggo, P.A., and Löbrich, M. (2009). ATM and Artemis promote homologous recombination of radiation-induced DNA double-strand breaks in G2. EMBO J. *28*, 3413–3427.

Broderick, R., Nieminuszczy, J., Baddock, H.T., Deshpande, R.A., Gileadi, O., Paull, T.T., McHugh, P.J., and Niedzwiedz, W. (2016). EXD2 promotes homologous recombination by facilitating DNA end resection. Nat. Cell Biol. *18*, 271–280.

Chang, H.H., Watanabe, G., and Lieber, M.R. (2015). Unifying the DNA end-processing roles of the artemis nuclease: Ku-dependent artemis resection at blunt DNA ends. J. Biol. Chem. *290*, 24036–24050.

Chanut, P., Britton, S., Coates, J., Jackson, S.P., and Calsou, P. (2016). Coordinated nuclease activities counteract Ku at single-ended DNA double-strand breaks. Nat. Commun. *7*, 12889.

Chapman, J.R., Barral, P., Vannier, J.B., Borel, V., Steger, M., Tomas-Loba, A., Sartori, A.A., Adams, I.R., Batista, F.D., and Boulton, S.J. (2013). RIF1 is essential for 53BP1-dependent nonhomologous end joining and suppression of DNA double-strand break resection. Mol. Cell *49*, 858–871.

DiBiase, S.J., Zeng, Z.C., Chen, R., Hyslop, T., Curran, W.J., Jr., and Iliakis, G. (2000). DNA-dependent protein kinase stimulates an independently active, nonhomologous, end-joining apparatus. Cancer Res. *60*, 1245–1253.

Escribano-Díaz, C., Orthwein, A., Fradet-Turcotte, A., Xing, M., Young, J.T., Tkáč, J., Cook, M.A., Rosebrock, A.P., Munro, M., Canny, M.D., et al. (2013). A cell cycle-dependent regulatory circuit composed of 53BP1-RIF1 and BRCA1-CtIP controls DNA repair pathway choice. Mol. Cell *49*, 872–883.

Feng, L., Fong, K.W., Wang, J., Wang, W., and Chen, J. (2013). RIF1 counteracts BRCA1-mediated end resection during DNA repair. J. Biol. Chem. *288*, 11135–11143.

Ghezraoui, H., Piganeau, M., Renouf, B., Renaud, J.B., Sallmyr, A., Ruis, B., Oh, S., Tomkinson, A.E., Hendrickson, E.A., Giovannangeli, C., et al. (2014). Chromosomal translocations in human cells are generated by canonical nonhomologous end-joining. Mol. Cell *55*, 829–842.

Goodarzi, A.A., Noon, A.T., Deckbar, D., Ziv, Y., Shiloh, Y., Löbrich, M., and Jeggo, P.A. (2008). ATM signaling facilitates repair of DNA double-strand breaks associated with heterochromatin. Mol. Cell *31*, 167–177.

Gotoh, E., and Durante, M. (2006). Chromosome condensation outside of mitosis: mechanisms and new tools. J. Cell. Physiol. *209*, 297–304.

Guirouilh-Barbat, J., Huck, S., Bertrand, P., Pirzio, L., Desmaze, C., Sabatier, L., and Lopez, B.S. (2004). Impact of the KU80 pathway on NHEJ-induced genome rearrangements in mammalian cells. Mol. Cell *14*, 611–623.

Huertas, P., and Jackson, S.P. (2009). Human CtIP mediates cell cycle control of DNA end resection and double strand break repair. J. Biol. Chem. *284*, 9558–9565.

Jackson, S.P., and Bartek, J. (2009). The DNA-damage response in human biology and disease. Nature *461*, 1071–1078.

Jette, N., and Lees-Miller, S.P. (2015). The DNA-dependent protein kinase: A multifunctional protein kinase with roles in DNA double strand break repair and mitosis. Prog. Biophys. Mol. Biol. *117*, 194–205.

Kakarougkas, A., Ismail, A., Katsuki, Y., Freire, R., Shibata, A., and Jeggo, P.A. (2013). Co-operation of BRCA1 and POH1 relieves the barriers posed by 53BP1 and RAP80 to resection. Nucleic Acids Res. *41*, 10298–10311.

Lansing, T.J., McConnell, R.T., Duckett, D.R., Spehar, G.M., Knick, V.B., Hassler, D.F., Noro, N., Furuta, M., Emmitte, K.A., Gilmer, T.M., et al. (2007). In vitro biological activity of a novel small-molecule inhibitor of polo-like kinase 1. Mol. Cancer Ther. *6*, 450–459.

Lee, B.I., and Wilson, D.M., 3rd (1999). The RAD2 domain of human exonuclease 1 exhibits 5′ to 3′ exonuclease and flap structure-specific endonuclease activities. J. Biol. Chem. *274*, 37763–37769.

Lee-Theilen, M., Matthews, A.J., Kelly, D., Zheng, S., and Chaudhuri, J. (2011). CtIP promotes microhomology-mediated alternative end joining during class-switch recombination. Nat. Struct. Mol. Biol. *18*, 75–79.

Leuther, K.K., Hammarsten, O., Kornberg, R.D., and Chu, G. (1999). Structure of DNA-dependent protein kinase: implications for its regulation by DNA. EMBO J. *18*, 1114–1123.

Lukas, J., and Lukas, C. (2013). Molecular biology. Shielding broken DNA for a quick fix. Science *339*, 652–653.

Ma, Y., Pannicke, U., Schwarz, K., and Lieber, M.R. (2002). Hairpin opening and overhang processing by an Artemis/DNA-dependent protein kinase complex in nonhomologous end joining and V(D)J recombination. Cell *108*, 781–794.

McVey, M., and Lee, S.E. (2008). MMEJ repair of double-strand breaks (director's cut): deleted sequences and alternative endings. Trends Genet. *24*, 529–538.

Moynahan, M.E., and Jasin, M. (2010). Mitotic homologous recombination maintains genomic stability and suppresses tumorigenesis. Nat. Rev. Mol. Cell Biol. *11*, 196–207.

Nishitani, H., Taraviras, S., Lygerou, Z., and Nishimoto, T. (2001). The human licensing factor for DNA replication Cdt1 accumulates in G1 and is destabilized after initiation of S-phase. J. Biol. Chem. *276*, 44905–44911.

Nussenzweig, A., and Nussenzweig, M.C. (2007). A backup DNA repair pathway moves to the forefront. Cell *131*, 223–225.

Ochi, T., Blackford, A.N., Coates, J., Jhujh, S., Mehmood, S., Tamura, N., Travers, J., Wu, Q., Draviam, V.M., Robinson, C.V., et al. (2015). DNA repair. PAXX, a paralog of XRCC4 and XLF, interacts with Ku to promote DNA double-strand break repair. Science *347*, 185–188.

Ochs, F., Somyajit, K., Altmeyer, M., Rask, M.B., Lukas, J., and Lukas, C. (2016). 53BP1 fosters fidelity of homology-directed DNA repair. Nat. Struct. Mol. Biol. *23*, 714–721.

Orthwein, A., Noordermeer, S.M., Wilson, M.D., Landry, S., Enchev, R.I., Sherker, A., Munro, M., Pinder, J., Salsman, J., Dellaire, G., et al. (2015). A mechanism for the suppression of homologous recombination in G1 cells. Nature *528*, 422–426.

Paull, T.T., and Gellert, M. (1998). The 3′ to 5′ exonuclease activity of Mre 11 facilitates repair of DNA double-strand breaks. Mol. Cell *1*, 969–979.

Rass, E., Grabarz, A., Plo, I., Gautier, J., Bertrand, P., and Lopez, B.S. (2009). Role of Mre11 in chromosomal nonhomologous end joining in mammalian cells. Nat. Struct. Mol. Biol. *16*, 819–824.

Reczek, C.R., Szabolcs, M., Stark, J.M., Ludwig, T., and Baer, R. (2013). The interaction between CtIP and BRCA1 is not essential for resection-mediated DNA repair or tumor suppression. J. Cell Biol. *201*, 693–707.

Riballo, E., Kühne, M., Rief, N., Doherty, A., Smith, G.C., Recio, M.J., Reis, C., Dahm, K., Fricke, A., Krempler, A., et al. (2004). A pathway of double-strand break rejoining dependent upon ATM, Artemis, and proteins locating to gamma-H2AX foci. Mol. Cell *16*, 715–724.

Sartori, A.A., Lukas, C., Coates, J., Mistrik, M., Fu, S., Bartek, J., Baer, R., Lukas, J., and Jackson, S.P. (2007). Human CtIP promotes DNA end resection. Nature *450*, 509–514.

Shakya, R., Reid, L.J., Reczek, C.R., Cole, F., Egli, D., Lin, C.S., deRooij, D.G., Hirsch, S., Ravi, K., Hicks, J.B., et al. (2011). BRCA1 tumor suppression depends on BRCT phosphoprotein binding, but not its E3 ligase activity. Science *334*, 525–528.

Shibata, A., Conrad, S., Birraux, J., Geuting, V., Barton, O., Ismail, A., Kakarougkas, A., Meek, K., Taucher-Scholz, G., Löbrich, M., and Jeggo, P.A. (2011). Factors determining DNA double-strand break repair pathway choice in G2 phase. EMBO J. *30*, 1079–1092.

Shibata, A., Moiani, D., Arvai, A.S., Perry, J., Harding, S.M., Genois, M.M., Maity, R., van Rossum-Fikkert, S., Kertokalio, A., Romoli, F., et al. (2014). DNA double-strand break repair pathway choice is directed by distinct MRE11 nuclease activities. Mol. Cell *53*, 7–18.

Turchi, J.J., Henkels, K.M., and Zhou, Y. (2000). Cisplatin-DNA adducts inhibit translocation of the Ku subunits of DNA-PK. Nucleic Acids Res. *28*, 4634–4641.

Wang, H., Rosidi, B., Perrault, R., Wang, M., Zhang, L., Windhofer, F., and Iliakis, G. (2005). DNA ligase III as a candidate component of backup pathways of nonhomologous end joining. Cancer Res. *65*, 4020–4030.

Williams, D.R., Lee, K.J., Shi, J., Chen, D.J., and Stewart, P.L. (2008). Cryo-EM structure of the DNA-dependent protein kinase catalytic subunit at subnanometer resolution reveals alpha helices and insight into DNA binding. Structure *16*, 468–477.

Williams, G.J., Hammel, M., Radhakrishnan, S.K., Ramsden, D., Lees-Miller, S.P., and Tainer, J.A. (2014). Structural insights into NHEJ: building up an integrated picture of the dynamic DSB repair super complex, one component and interaction at a time. DNA Repair (Amst.) *17*, 110–120.

Wu, J., Lu, L.Y., and Yu, X. (2010). The role of BRCA1 in DNA damage response. Protein Cell *1*, 117–123.

Yu, X., and Chen, J. (2004). DNA damage-induced cell cycle checkpoint control requires CtIP, a phosphorylation-dependent binding partner of BRCA1 C-terminal domains. Mol. Cell. Biol. *24*, 9478–9486.

Yu, X., Fu, S., Lai, M., Baer, R., and Chen, J. (2006). BRCA1 ubiquitinates its phosphorylation-dependent binding partner CtIP. Genes Dev. *20*, 1721–1726.

Yun, M.H., and Hiom, K. (2009). CtIP-BRCA1 modulates the choice of DNA double-strand-break repair pathway throughout the cell cycle. Nature *459*, 460–463.

Zhang, Y., and Jasin, M. (2011). An essential role for CtIP in chromosomal translocation formation through an alternative end-joining pathway. Nat. Struct. Mol. Biol. *18*, 80–84.

STAR★METHODS

KEY RESOURCES TABLE

REAGENT or RESOURCE	SOURCE	IDENTIFIER
Antibodies		
Rabbit-anti-53BP1	Bethyl	A300-272A
Mouse-anti-53BP1 (clone BP13)	Millipore	#05-726
Rabbit-anti-Artemis	Novus Biologicals	NB100-542
Rabbit-anti-Artemis	GenTex	GTX100128
Rabbit-anti-Artemis	Abcam	ab35649
Rabbit-anti-BLM	Abcam	ab2179
Mouse-anti-Brca1 (D-9)	Santa Cruz	sc-6954
Rabbit-anti-Brca1 (C-20)	Santa Cruz	sc-642
Mouse-anti-Brca1_MS13	Abcam	ab16781
Mouse-anti-BrdU (3D4)	BD PharMingen	555627
Mouse-anti-CD4-FITC	Biolegend	100510
Mouse-anti-CtIP (E-2)	Santa Cruz	sc-48415
Rabbit-anti-CtIP	Bethyl	A300-488A
Mouse-anti-CtIP (D-4)	Santa Cruz	sc-271339
Rabbit-anti-pCtIP (Ser327)	Phosphosolutions	N/A
Rabbit-anti-CDT1	Abcam	ab202067
Rabbit-anti-DNA2	Abcam	ab 96488
Rabbit-anti-DNA-PKcs	Novus Biologicals	NB100-658
Rabbit-anti-EXD2	Sigma	HPA005848
Mouse-anti-Exo1	Abcam	ab3307
Mouse-anti-Flag (M2)	Sigma	F3165
Rabbit-anti-GAPDH (FL-335)	Santa Cruz	sc-25778
Mouse-anti-GFP	Roche	11 814 460 001
Rabbit-anti-GFP	Santa Cruz	sc-8334
Mouse-anti-phospho-Histone H2A.X (Ser139)	Millipore	# 05-636
Rabbit-anti-phospho-Histone H2A.X (Ser139)	Abcam	ab81299
Mouse-anti-HA tag (HA.C5)	Abcam	ab18181
Mouse-anti-Ku70 (A-9)	Santa Cruz	sc-5309
Mouse-anti-Ku80 (111)	Abcam	ab79220
Mouse-anti-Lig1 (1A9)	Santa Cruz	sc-47703
Mouse-anti-Lig3	Santa Cruz	sc-56089
Rabbit-anti-Lig4	Acris	SP1275
Mouse-anti-cMyc (9E10)	Santa Cruz	sc-40
Rabbit-anti-Plk3	Abcam	ab33119
Rabbit-anti-tRFP	Evrogen	AB233
Rabbit-anti-RPA32/RPA2 (phosphoT21)	Abcam	ab109394
Rabbit-anti-I-SceI (FL-86)	Santa Cruz	sc-98269
Mouse-anti-alpha-Tubulin (TU-02)	Santa Cruz	sc- 8035
Goat-anti-mouse IgG-HRP	Santa Cruz	sc-2031
Goat-anti-rabbit IgG-HRP	Santa Cruz	sc-2030
Goat-anti-mouse IgG AlexaFluor 488	Molecular Probes	A11001
Goat-anti-mouse IgG AlexaFluor 594	Molecular Probes	A11005
Goat-anti-rabbit IgG AlexaFluor 488	Molecular Probes	A11008
Goat-anti-rabbit IgG AlexaFluor 594	Molecular Probes	A11012

(*Continued on next page*)

Continued

REAGENT or RESOURCE	SOURCE	IDENTIFIER
Chemicals, Peptides, and Recombinant Proteins		
Plk inhibitor GW 843682X	Tocris Bioscience	2977
DNA-PK inhibitor Nu7441	Tocris Bioscience	3712
EdU	baseclick	BCN-001
BrdU	BD Bioscience	550891
DAPI	Sigma-Aldrich	D9542
PARP inhibitor PJ34	Calbiochem	528151
Mre11 (endo) inhibitor (PFM01)	Shibata et al., 2014	N/A
Mre11 (exo) inhibitor (PFM39)	Shibata et al., 2014	N/A
Dynabeads Protein G	Thermo Scientific	10004D
Anti-rat IgG MicroBeads	Miltenyi Biotec	130-048-501
Nocodazole	Sigma-Aldrich	M1404
KaryoMAX Colcemid	GIBCO	15212012
Polyethylenglycol (PEG)	Roche	10783641001
RNase-A	Sigma-Aldrich	R4875
Thymidine	Sigma-Aldrich	T1895-1G
KOD Hot Start DNA Polymerase	Novagen	71086-4
Critical Commercial Assays		
Effectene Transfection Reagent	QIAGEN	301425
jetPEI Transfection Reagent	Polyplus	13-101-10
HiPerFect Transfection Reagent	QIAGEN	301707
PEI	Sigma-Aldrich	408727-7
EdU-Click Kit (Cy5)	baseclick	BCK-EDU-647-1
peqGOLD Xchange Plasmid maxi-EF Kit	peqlab	12-7404-01
MACS separation column	Miltenyi Biotec	130-042-201
MasterPure Complete DNA & RNA Purification	Epicenter	MC85200
PureLink Genomic DNA Mini Kit	Thermo Scientific	K1820-01
FISH XCP Mix	MetaSystems	D-0328-200-MC
LumiLight Western Blotting Substrate	Roche	12015200001
WesternBright Quantum	Advansta	541015
WesternBright Sirius	Advansta	541021
Experimental Models: Cell Lines		
Human: 82-6 hTert	Riballo et al., 2004	N/A
Human: CJ179 hTert	Riballo et al., 2004	N/A
Human: HeLa-S3	ATCC	ATCC-CCL-2.2
Human: GC92	Rass et al., 2009	N/A
Mouse: MEF Brca1-wt	Shakya et al., 2011	N/A
Mouse: MEF Brca1-ΔBRCT	Shakya et al., 2011	N/A
Human: 2BN hTert	Shibata et al., 2011	N/A
Human: 411BR hTert	Jeggo Lab (Sussex)	N/A
Human: HeLa pGC	Barton et al., 2014	N/A
Experimental Models: Organisms/Strains		
DH5α *E. coli*	This paper	N/A
Recombinant DNA		
pGEM-T Easy Vector	Promega	A1360
pUC19	New England Biolabs	N3041S
pEGFP_C1	Clontech	632470
tRFP	Jeggo Lab (Sussex)	N/A

(*Continued on next page*)

Continued		
REAGENT or RESOURCE	SOURCE	IDENTIFIER
siRNA-resistant pEGFP-CtIP	Barton et al., 2014	N/A
siRNA-resistant pCIneo-cMyc-Artemis	Beucher et al., 2009	N/A
siRNA-resistant Flag-Plk3	Barton et al., 2014	N/A
siRNA-resistant Flag-Brca1	Shakya et al., 2011	N/A
siRNA-resistant HA-53BP1	Jeggo Lab (Sussex)	N/A
siRNA-resistant Flag-Exo1	Jeggo Lab (Sussex)	N/A
Sequence-Based Reagents		
see Table S2		
Software and Algorithms		
Metafer	MetaSystems	N/A
LAS AF Lite	Leica	N/A
AxioVision V4.6.3.0	Zeiss Imaging Solutions	N/A
ImageJ	Open Source	N/A
ChemiCapt	Vilber Lourmat	N/A
FusionCapt Advance FX7	Vilber Lourmat	N/A
Other		
X-ray tube: MCN 165/796704	Philips	N/A
Microscope: Axiovert 200M	Zeiss	N/A
Microscope: Image Z.2	Zeiss	N/A
Confocal laser scanning microscope: TCS SP5 II	Leica	N/A
Chemiluminescence detection: ChemiSmart 5000	Vilber Lourmat	N/A
Chemiluminescence detection: Fusion FX	Vilber Lourmat	N/A

CONTACT FOR REAGENT AND RESOURCE SHARING

Further information and requests for reagents may be directed to, and will be fulfilled by the Lead Contact, Markus Löbrich (lobrich@bio.tu-darmstadt.de).

EXPERIMENTAL MODEL AND SUBJECT DETAILS

Cell Lines and Cell Culture

Cell lines used were control 82-6 hTert (Riballo et al., 2004), Artemis-deficient CJ179 hTert (Riballo et al., 2004), HeLa (ATCC), GC92 (Rass et al., 2009), Brca1-wt and Brca1-ΔBRCT MEFs (Shakya et al., 2011), XLF-deficient 2BN hTert (Shibata et al., 2011), hypomorphic Lig4-mutated 411Br hTert (Jeggo Lab), and HeLa pGC (Barton et al., 2014). Cells were tested for mycoplasm contamination by PCR, HeLa cells were authenticated by ATCC. HeLa, GC92 cells and MEFs were cultured in DMEM with 10% FCS and 1% NEAA, 82-6, CJ179, 411Br and 2BN cells in MEM with 20% FCS, 1% NEAA. All cells were maintained at 37°C in a 5% CO_2 incubator.

Bacterial Strains

Competent DH5α *E.coli* were used for transformations.

METHOD DETAILS

Generation of Artemis KO Cells with CRISPR/Cas9

Vectors encoding Artemis guide RNAs (see Key Resources Table) were used. GC92 cells were transfected with the Artemis gRNA plasmids, a Cas9 and a EGFP plasmid using PEI following the manufacturer's instructions. Single GFP-positive cells were sorted into 96 well plates and tested for knockdown on a protein level using immunoblotting. Genomic DNA was extracted from potential KO cells with PureLink Genomic DNA Mini Kit and PCR was performed with KOD Hot Start DNA Polymerase to amplify the targeted regions. PCR products were cloned into pGEM-T Easy Vector and transformed into DH5α competent *E. coli*. Isolated plasmid DNA of at least 10 colonies from each transformation were sent for sequencing to ensure frameshift mutation in the targeted region.

RNA Interference and Plasmid Transfection

SiRNA transfection of HeLa, 82-6, CJ179, and GC92 cells and MEFs was carried out using HiPerFect Transfection Reagent following the manufacturer's instructions. 53BP1 (25nM), Artemis (15 nM), BLM (50nM), Brca1 (25 nM), CtIP (50 nM), DNA2 (20nM), DNA-PKcs (15 nM), EXD2 (25nM), Exo1 (20 nM), Ku70 (25 nM), Ku80 (25 nM), Lig1 (25 nM), Lig3 (25 nM), Lig4 (20 nM), and Plk3 (25 nM) siRNAs were used (target sequences are listed in the Key Resources Table). Experiments were either performed 48 hr after transfection or after 72 hr with an additional siRNA transfection after 24 hr. In complementation studies, the endogenous protein was depleted by siRNA in HeLa, MEF, 82-6, CJ179 or GC92 cells and 24 hr after siRNA transfection, cells were transfected with various plasmids (see Key Resources Table) using Effectene or jetPEI following the manufacturer's instructions. HeLa cells for immunoprecipitation experiments were transfected with PEI following the manufacturer's instructions. For I-SceI-transfection, GC92 or HeLa pGC cells were transfected using jetPEI following the manufacturer's instructions.

IR and Chemical Treatment

X-IR was performed at 90 kV and 19 mA. A ^{241}Am source was used for α-IR. Chemical inhibitors were added 1 hr prior to IR and maintained during repair incubation. The Plk inhibitor GW 843682X (IC_{50} values of 2.2 and 9.1 nM for Plk1 and Plk3, respectively), the DNA-PK inhibitor Nu7441, the PARP inhibitor PJ34, the Mre11 (endo) inhibitor and the Mre11 (exo) inhibitor (Shibata et al., 2014) were used at concentrations of 0.5, 7.5, 15, 50, and 300 μM.

Chromosomal Analysis

For the analysis of translocations and chromosome breaks in G1, exponentially growing or confluent 82-6 or CJ179 fibroblasts were irradiated. To prevent progression of G2-irradiated cells into G1 during repair incubation, cells were treated with nocodazole (100 ng/ml) prior to IR. After repair incubation, cells were mixed at a ratio of 1:1 with mitotic HeLa cells (enriched by treatment with colcemid for 20 h). Cell fusion was mediated by Polyethylenglycol (PEG). For FISH experiments whole chromosome probes 1, 2 and 4 were used and the staining was performed following the manufacturer's protocol (MetaSystems). Pictures of the chromosomes were acquired by using an Axioplan2 microscope with an EC Plan Neofluar (63x) (Zeiss) and Metafer software (MetaSystems). Only the stained chromosomes were analyzed.

Protein Extracts, Immunoprecipitation, and Immunoblotting

Knockdown efficiencies and expression of exogenous plasmids were confirmed by immunoblotting. For immunoprecipitation, 2 μg antibodies (see Key Resource Table) were linked to Dynabeads Protein G, washed three times in 0.1% BSA/PBS and then incubated with the cell extract at 4°C for 2 hr. After immunoblotting, the membrane was blocked in 5% low fat milk or 5% BSA in TBS/0.1% Tween20. Immunoblotting was carried out in TBS/0.1% Tween20/1% low fat milk or 5% BSA over night at 4°C, followed by HRP-conjugated secondary antibody incubation in PBS/0.1% Tween20/1% low fat milk or 5% BSA for 1 hr. Immunoblots were developed using LumiLight immunoblotting substrate or WesternBright Quantum or Sirius. Signal detection was performed with ChemiSmart5000 or Fusion FX. Antibodies are listed in the Key Resources Table.

Immunofluorescence

Cells were grown on glass coverslips for X-IR and on Mylar foil for α-IR. The thymidine analog EdU and nocodazole (100 ng/μl) were added 30 min prior to IR and cells were fixed and stained. EdU incorporation was detected with an EdU-Click kit. For BrdU foci analysis, cells were pre-extracted for 10 min with 0.5% Triton X-100. Cells were examined with a Zeiss microscope and Metafer software. For each foci counting experiment at least 40 cells were evaluated. For Ku80 foci staining, cells were pre-extracted and stained as previously described (Chanut et al., 2016). Additionally, after the secondary antibody staining, cells were fixed with 2% PFA for 10 min. For image acquisition Z stacks were obtained with a confocal laser scanning microscope using a 100x immersion objective. Co-localization of Ku80 and pRPA or Rad51 foci was analyzed using LAS AF Lite software (dx < 20 nm for co-localization of signal intensity in line profile). Antibodies are listed in the Key Resources Table.

Reporter Assays

HeLa pGC cells containing an HR substrate were transfected with a I-SceI-plasmid 24 hr after seeding and treated with inhibitors. GC92 cells containing an NHEJ substrate were transfected with siRNA. For complementation studies, cells were transfected with constructs 24 hr after seeding. 48 hr after seeding, cells were transfected with a I-SceI-expressing plasmid and either treated with an inhibitor or transfected again with siRNA. After 72 hr the cells were fixed and stained. Up to 10,000 cells were analyzed per sample for either GFP (HeLa pGC cells) or CD4 (for GC92) positive cells with a microscope (Axiovert 200M) and Metafer software. Antibodies are listed in the Key Resources Table.

Sequence Analysis in NHEJ Reporter Assay

GC92 cells were dissociated with 50 mM EDTA in PBS and stained with 1.5 μg rat-α-CD4-FITC antibody for 30 min at 4°C. After washing, cells were incubated 15 min at 4°C with goat-α-rat microbeads. Then, the CD4-positive GC92 cells were separated and

enriched by using a miniMACS column. After purification of DNA with MasterPure Complete DNA & RNA Purification, PCR was performed using the primers listed in the Key Resources Table. To isolate the individual clones, PCR products were cloned into pUC19 and sequenced (MWG Eurofins).

Anti-pSer327 Antibody Preparation

Phospho-specific antibodies were produced in rabbits against CtIP-pSer327 (custom antibody service from Phosphosolutions). The antigens were synthetic phospho-peptides corresponding to amino acids surrounding the phosphorylated Ser327 in the human CtIP sequence. The resulting solution of purified antibody was stored at -20°C.

Cell Synchronization and Flow Cytometry

Proliferating HeLa cells were treated with 2 mM thymidine for 16 hr, released in thymidine-free medium for 10 hr and again pulse-treated with thymidine for 14 hr. Cells were again released in fresh medium without thymidine for 18 hr to obtain G1 cells. Cell synchronization was controlled by propidium iodide and BrdU flow cytometry analysis.

QUANTIFICATION AND STATISTICAL ANALYSIS

All data were derived from at least n = 3 replicates for foci analysis and chromosomal studies, or from at least n = 4 biological replicates for the NHEJ and HR reporter assays. Column plots show the mean value and boxplots were created with SigmaPlot12.0. Background foci/chromosome breaks/chromosome translocations were subtracted from the mean values. The error bars in the column plots show the SEM between the experiments. p values in column and line plots originate from Student's t test. They compare all cells analyzed in foci and chromosomal experiments and compare the mean data in the NHEJ and HR reporter assay (*, $p < 0.05$; **, $p < 0.01$; ***, $p < 0.001$). p values in boxplots originate from Mann-Whitney U test analyzed with SigmaPlot12.0.

Molecular Cell, Volume *65*

Supplemental Information

DNA Double-Strand Break Resection Occurs during Non-homologous End Joining in G1 but Is Distinct from Resection during Homologous Recombination

Ronja Biehs, Monika Steinlage, Olivia Barton, Szilvia Juhász, Julia Künzel, Julian Spies, Atsushi Shibata, Penny A. Jeggo, and Markus Löbrich

Figure S1

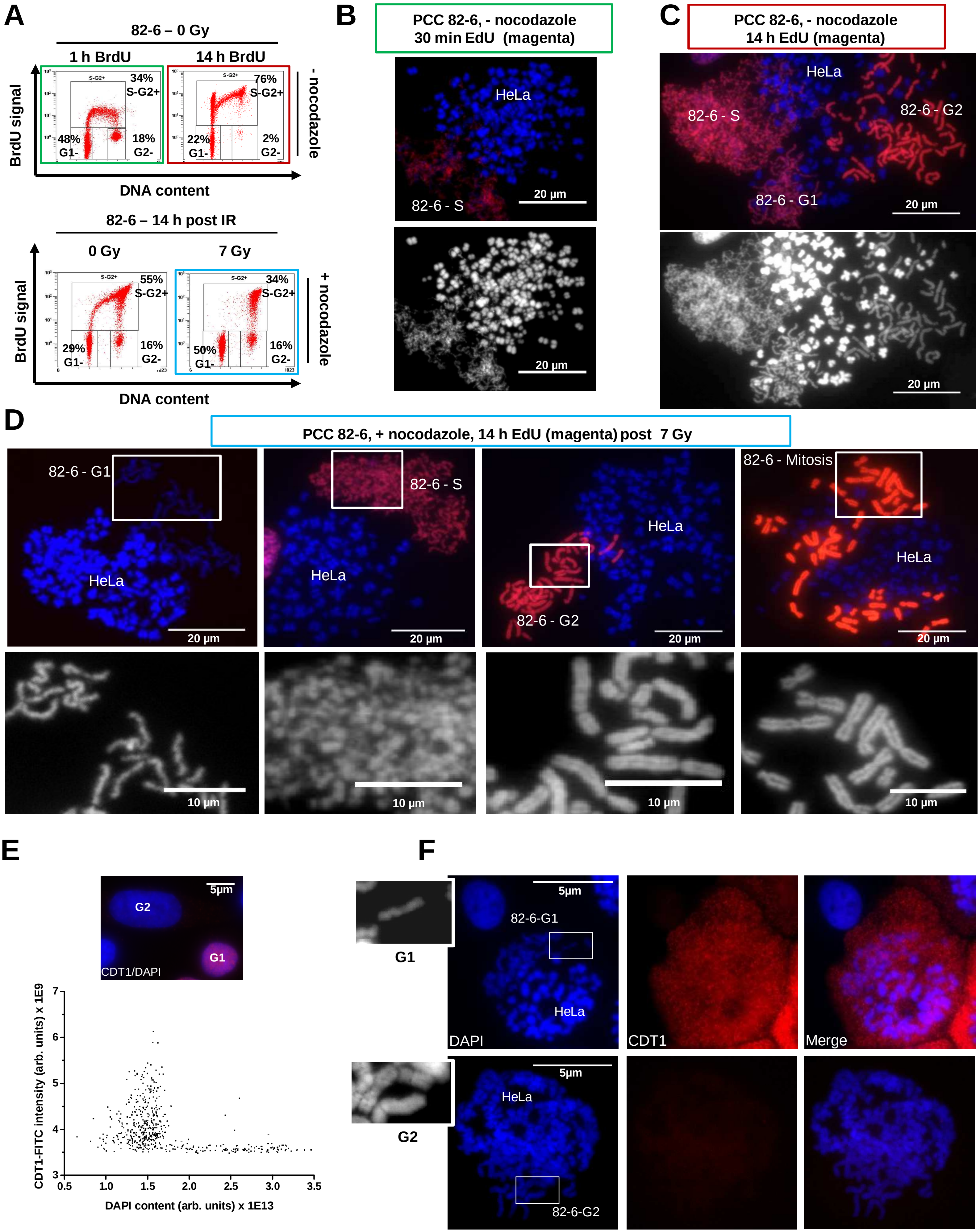
A
82-6 – 0 Gy
1 h BrdU
14 h BrdU
BrdU signal
34%
S-G2+
48%
G1-
18%
G2-
76%
S-G2+
22%
G1-
2%
G2-
- nocodazole
DNA content
82-6 – 14 h post IR
0 Gy
7 Gy
55%
S-G2+
29%
G1-
16%
G2-
34%
S-G2+
50%
G1-
16%
G2-
+ nocodazole
B
PCC 82-6, - nocodazole
30 min EdU (magenta)
HeLa
82-6 - S
20 µm
C
PCC 82-6, - nocodazole
14 h EdU (magenta)
HeLa
82-6 - S
82-6 - G2
82-6 - G1
20 µm
D
PCC 82-6, + nocodazole, 14 h EdU (magenta) post 7 Gy
82-6 - G1
HeLa
82-6 - S
82-6 - G2
82-6 - Mitosis
20 µm
10 µm
E
G2
G1
5µm
CDT1/DAPI
CDT1-FITC intensity (arb. units) x 1E9
DAPI content (arb. units) x 1E13
F
G1
G2
5µm
82-6-G1
HeLa
82-6-G2
DAPI
CDT1
Merge

Figure S2

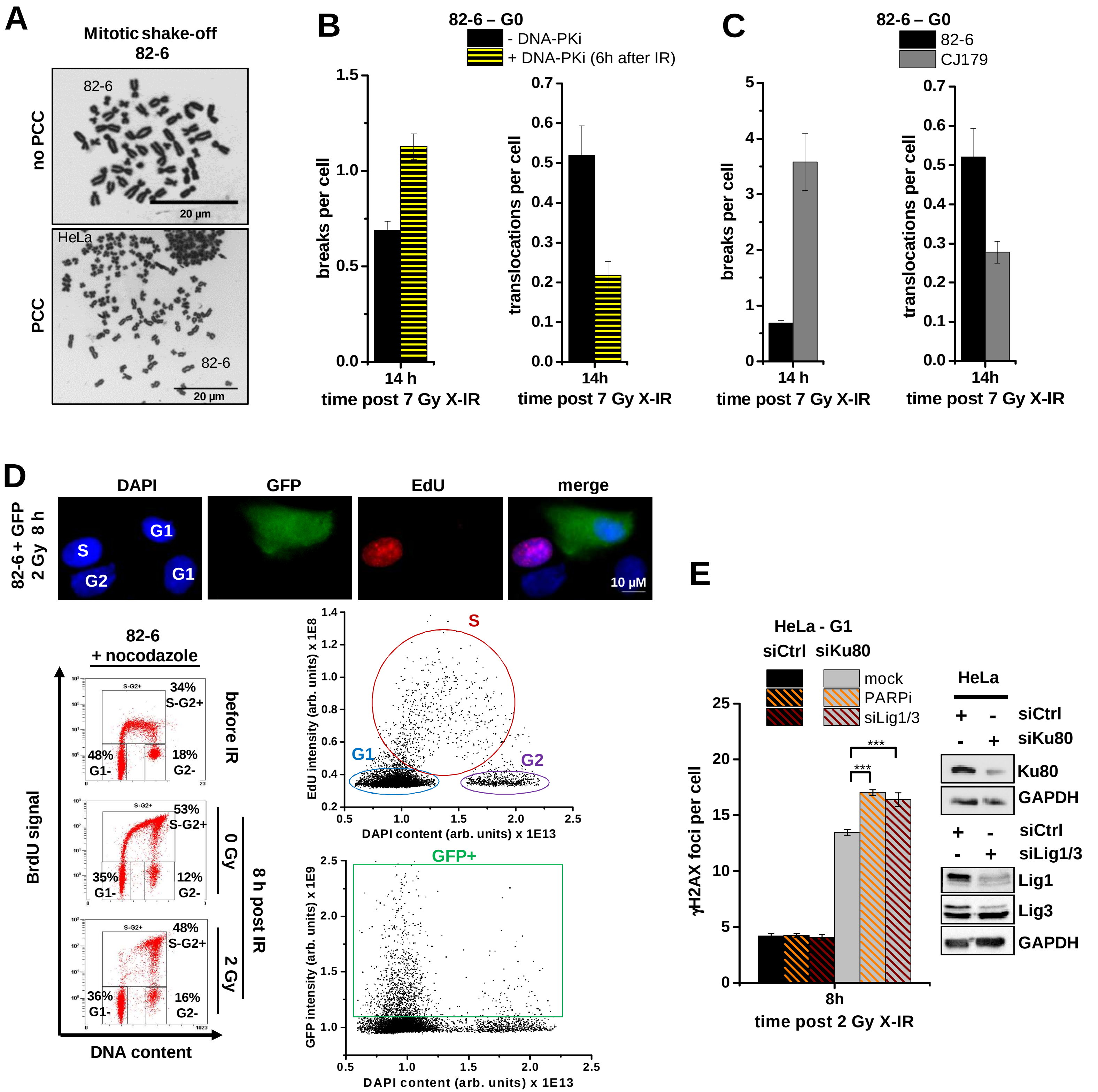

A
Mitotic shake-off 82-6
no PCC
PCC
82-6
HeLa
20 µm
B
82-6 – G0
- DNA-PKi
+ DNA-PKi (6h after IR)
breaks per cell
translocations per cell
14 h
14h
time post 7 Gy X-IR
C
82-6 – G0
82-6
CJ179
D
DAPI
GFP
EdU
merge
82-6 + GFP 2 Gy 8 h
S
G1
G2
10 µM
82-6 + nocodazole
BrdU signal
DNA content
before IR
0 Gy
2 Gy
8 h post IR
S-G2+
G1-
G2-
34%
48%
18%
53%
35%
12%
36%
16%
EdU intensity (arb. units) x 1E8
DAPI content (arb. units) x 1E13
GFP intensity (arb. units) x 1E9
GFP+
E
HeLa - G1
siCtrl siKu80
mock
PARPi
siLig1/3
γH2AX foci per cell
8h
time post 2 Gy X-IR
HeLa
siCtrl
siKu80
siLig1/3
Ku80
GAPDH
Lig1
Lig3

Figure S3

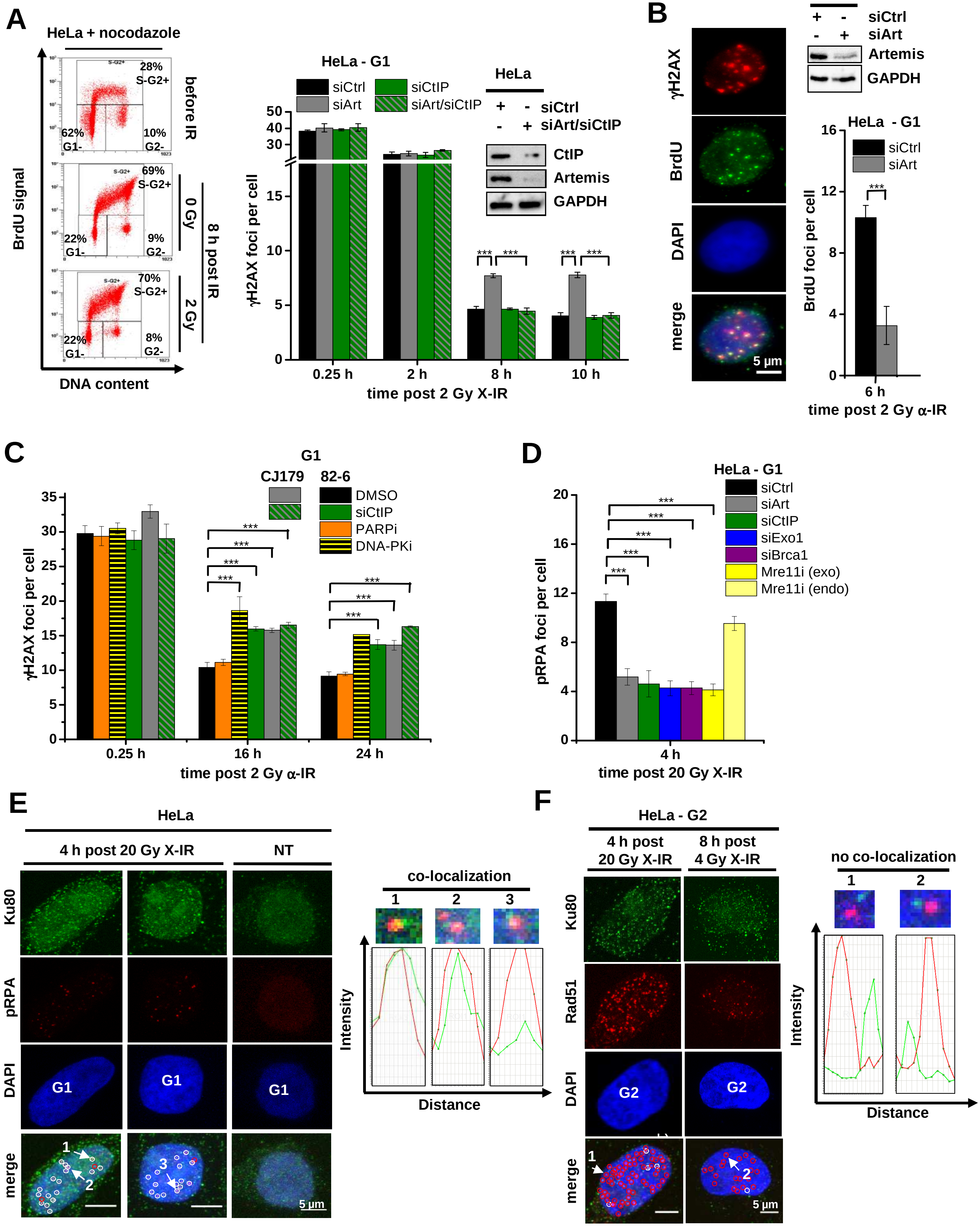

A
HeLa + nocodazole
BrdU signal
DNA content
before IR
0 Gy
2 Gy
8 h post IR
28% S-G2+
62% G1-
10% G2-
69% S-G2+
22% G1-
9% G2-
70% S-G2+
22% G1-
8% G2-
HeLa - G1
siCtrl
siArt
siCtIP
siArt/siCtIP
γH2AX foci per cell
0.25 h
2 h
8 h
10 h
time post 2 Gy X-IR
HeLa
siCtrl
siArt/siCtIP
CtIP
Artemis
GAPDH
B
γH2AX
BrdU
DAPI
merge
5 µm
HeLa
siCtrl
siArt
Artemis
GAPDH
HeLa - G1
BrdU foci per cell
6 h
time post 2 Gy α-IR
C
G1
CJ179
82-6
DMSO
siCtIP
PARPi
DNA-PKi
γH2AX foci per cell
0.25 h
16 h
24 h
time post 2 Gy α-IR
D
HeLa - G1
siCtrl
siArt
siCtIP
siExo1
siBrca1
Mre11i (exo)
Mre11i (endo)
pRPA foci per cell
4 h
time post 20 Gy X-IR
E
HeLa
4 h post 20 Gy X-IR
NT
Ku80
pRPA
DAPI
merge
G1
5 µm
co-localization
Intensity
Distance
F
HeLa - G2
4 h post 20 Gy X-IR
8 h post 4 Gy X-IR
Ku80
Rad51
DAPI
merge
G2
5 µm
no co-localization
Intensity
Distance

Figure S4

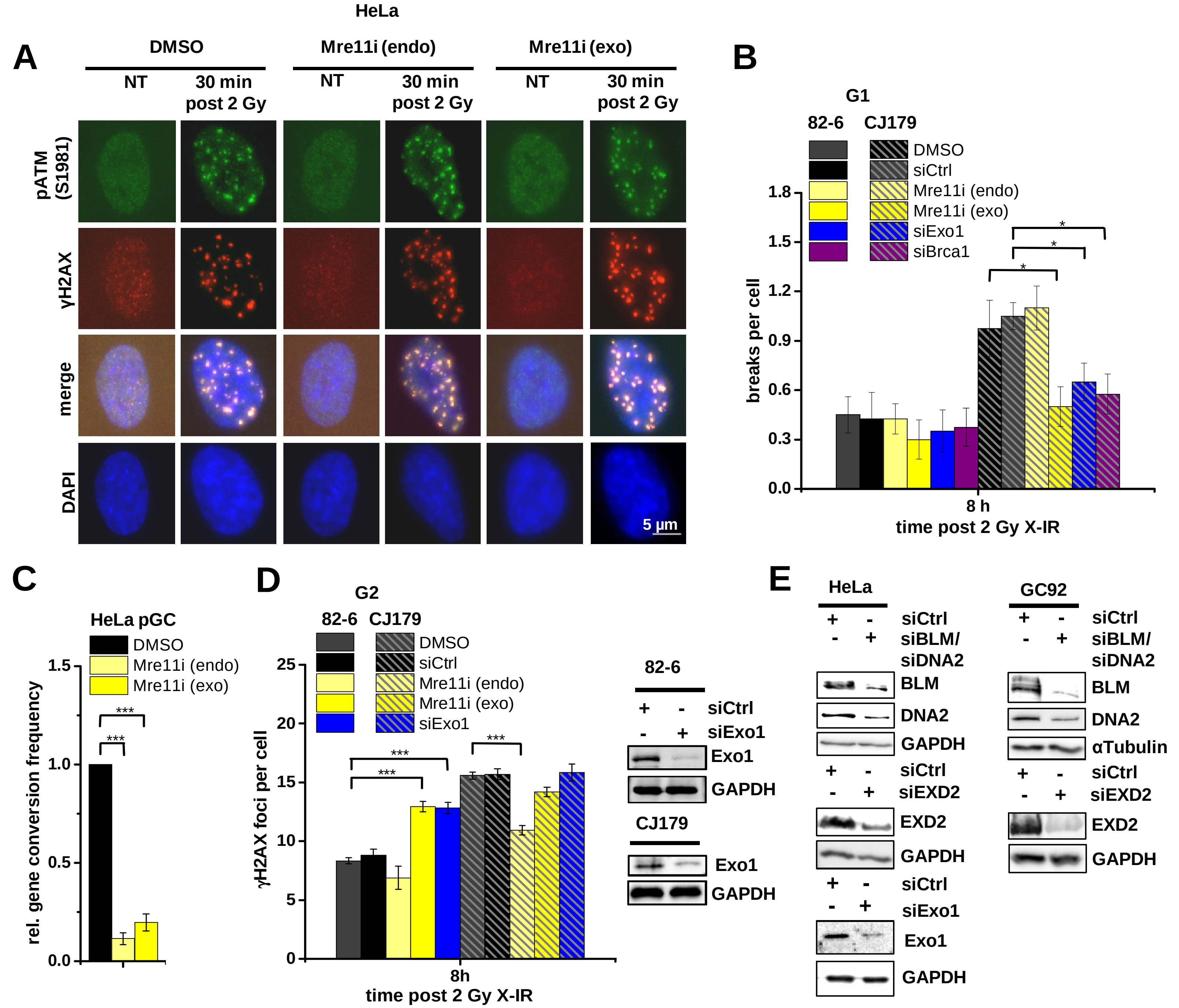

HeLa
A
DMSO
Mre11i (endo)
Mre11i (exo)
NT
30 min post 2 Gy
pATM (S1981)
γH2AX
merge
DAPI
5 µm
B
G1
82-6
CJ179
DMSO
siCtrl
Mre11i (endo)
Mre11i (exo)
siExo1
siBrca1
breaks per cell
8 h
time post 2 Gy X-IR
C
HeLa pGC
DMSO
Mre11i (endo)
Mre11i (exo)
rel. gene conversion frequency
D
G2
82-6
CJ179
DMSO
siCtrl
Mre11i (endo)
Mre11i (exo)
siExo1
γH2AX foci per cell
8h
time post 2 Gy X-IR
82-6
siCtrl
siExo1
Exo1
GAPDH
CJ179
Exo1
GAPDH
E
HeLa
siCtrl
siBLM/ siDNA2
BLM
DNA2
GAPDH
siCtrl
siEXD2
EXD2
GAPDH
siCtrl
siExo1
Exo1
GAPDH
GC92
siCtrl
siBLM/ siDNA2
BLM
DNA2
αTubulin
siCtrl
siEXD2
EXD2
GAPDH

Figure S5

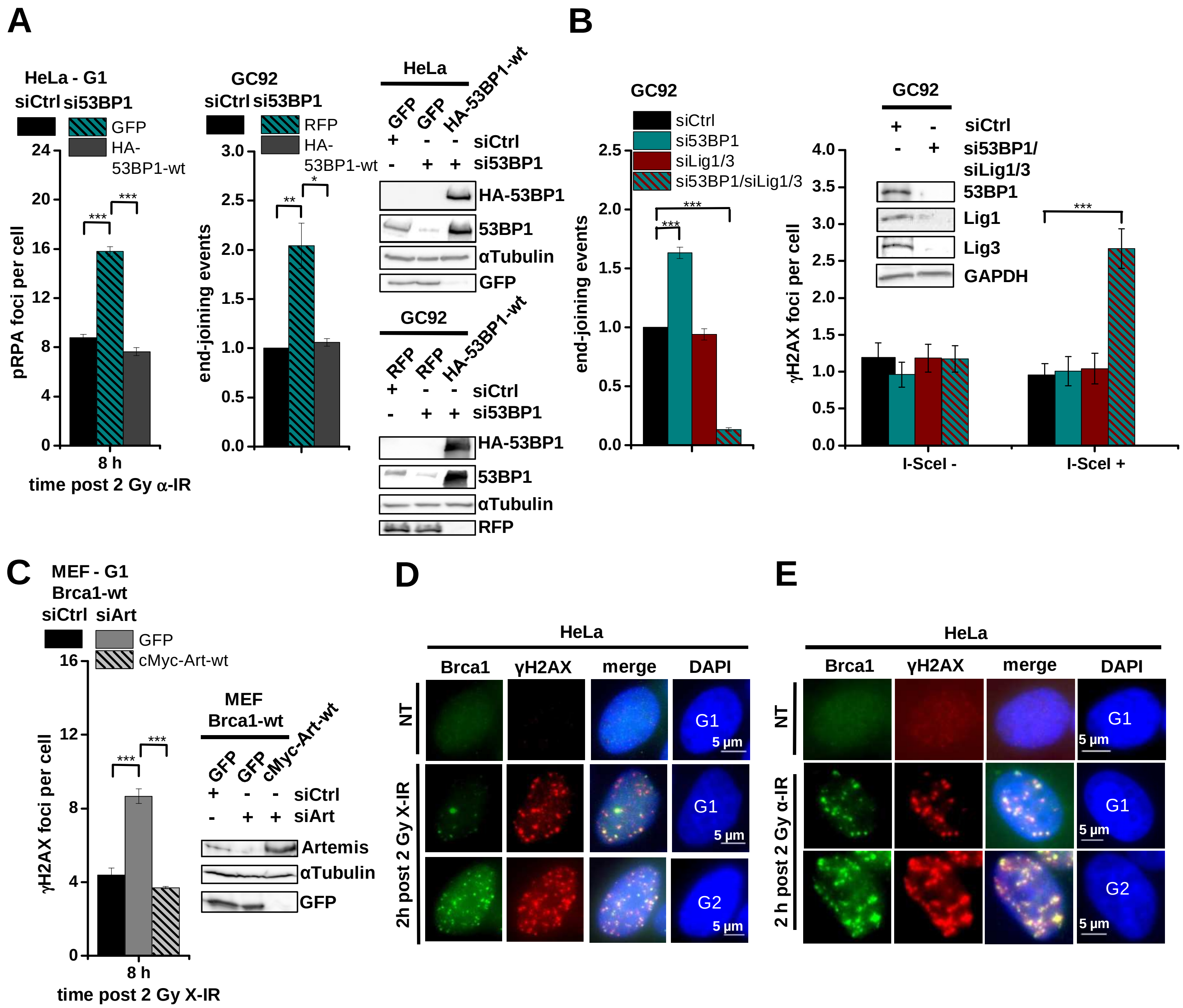
A
HeLa - G1
siCtrl si53BP1
GFP
HA-53BP1-wt
pRPA foci per cell
***
***
8 h
time post 2 Gy α-IR
GC92
siCtrl si53BP1
RFP
HA-53BP1-wt
end-joining events
**
*
HeLa
GFP GFP HA-53BP1-wt
+ - - siCtrl
- + + si53BP1
HA-53BP1
53BP1
αTubulin
GFP
GC92
RFP RFP HA-53BP1-wt
+ - - siCtrl
- + + si53BP1
HA-53BP1
53BP1
αTubulin
RFP
B
GC92
siCtrl
si53BP1
siLig1/3
si53BP1/siLig1/3
end-joining events
***
***
GC92
+ - siCtrl
- + si53BP1/siLig1/3
53BP1
Lig1
Lig3
GAPDH
γH2AX foci per cell
***
I-SceI -
I-SceI +
C
MEF - G1
Brca1-wt
siCtrl siArt
GFP
cMyc-Art-wt
γH2AX foci per cell
***
***
8 h
time post 2 Gy X-IR
MEF
Brca1-wt
GFP GFP cMyc-Art-wt
+ - - siCtrl
- + + siArt
Artemis
αTubulin
GFP
D
HeLa
Brca1 γH2AX merge DAPI
NT
2h post 2 Gy X-IR
G1
G1
G2
5 µm
E
HeLa
Brca1 γH2AX merge DAPI
NT
2 h post 2 Gy α-IR
G1
G1
G2
5 µm

Figure S6

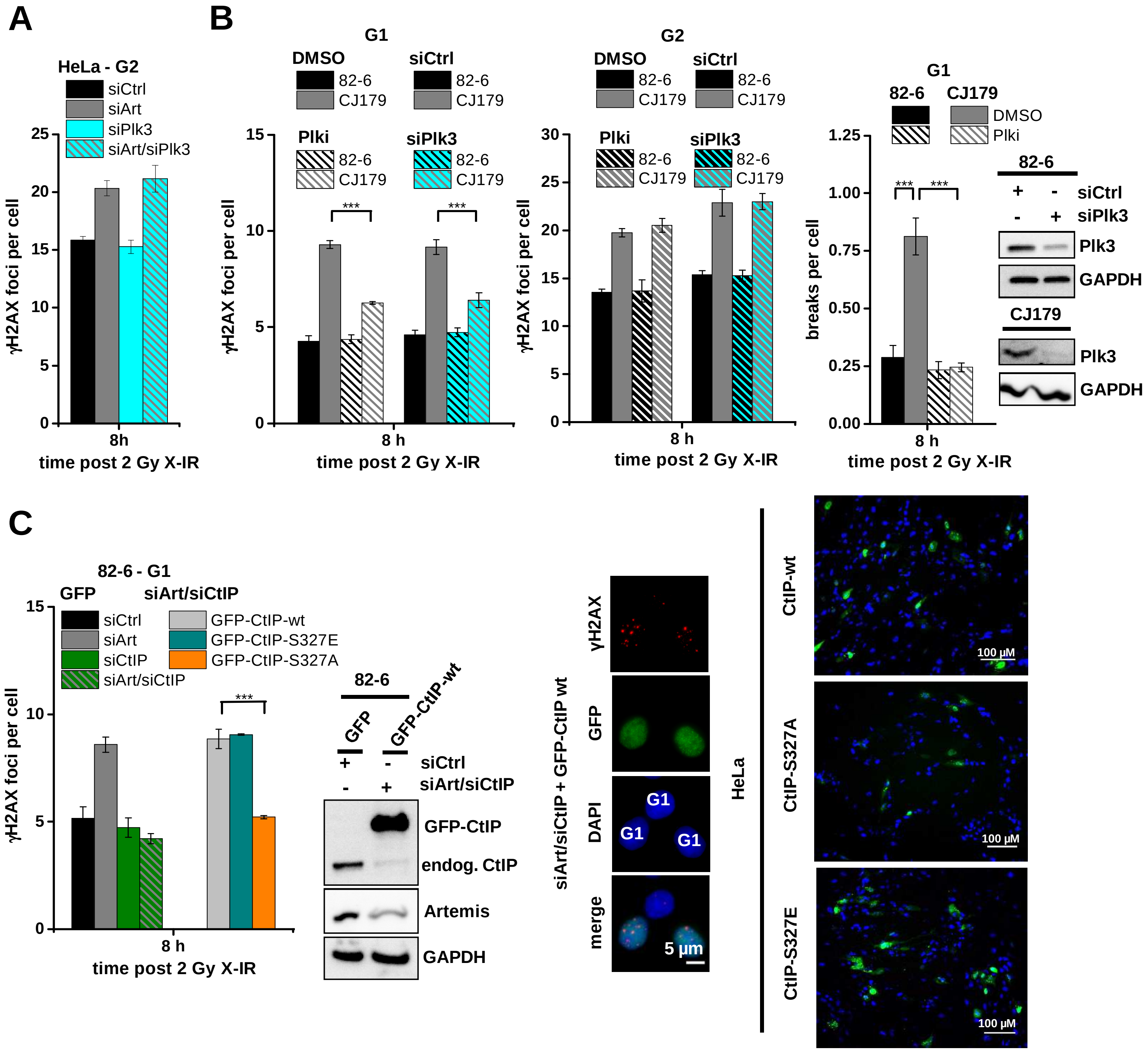

A
B
C
HeLa - G2
siCtrl
siArt
siPlk3
siArt/siPlk3
γH2AX foci per cell
8h
time post 2 Gy X-IR
G1
DMSO
siCtrl
82-6
CJ179
Plki
siPlk3
***
8 h
G2
breaks per cell
DMSO
Plki
siCtrl
siPlk3
Plk3
GAPDH
82-6 - G1
GFP
siArt/siCtIP
siCtIP
GFP-CtIP-wt
GFP-CtIP-S327E
GFP-CtIP-S327A
GFP-CtIP
endog. CtIP
Artemis
siArt/siCtIP + GFP-CtIP wt
γH2AX
DAPI
merge
5 µm
HeLa
CtIP-wt
CtIP-S327A
CtIP-S327E
100 µM

Figure S7

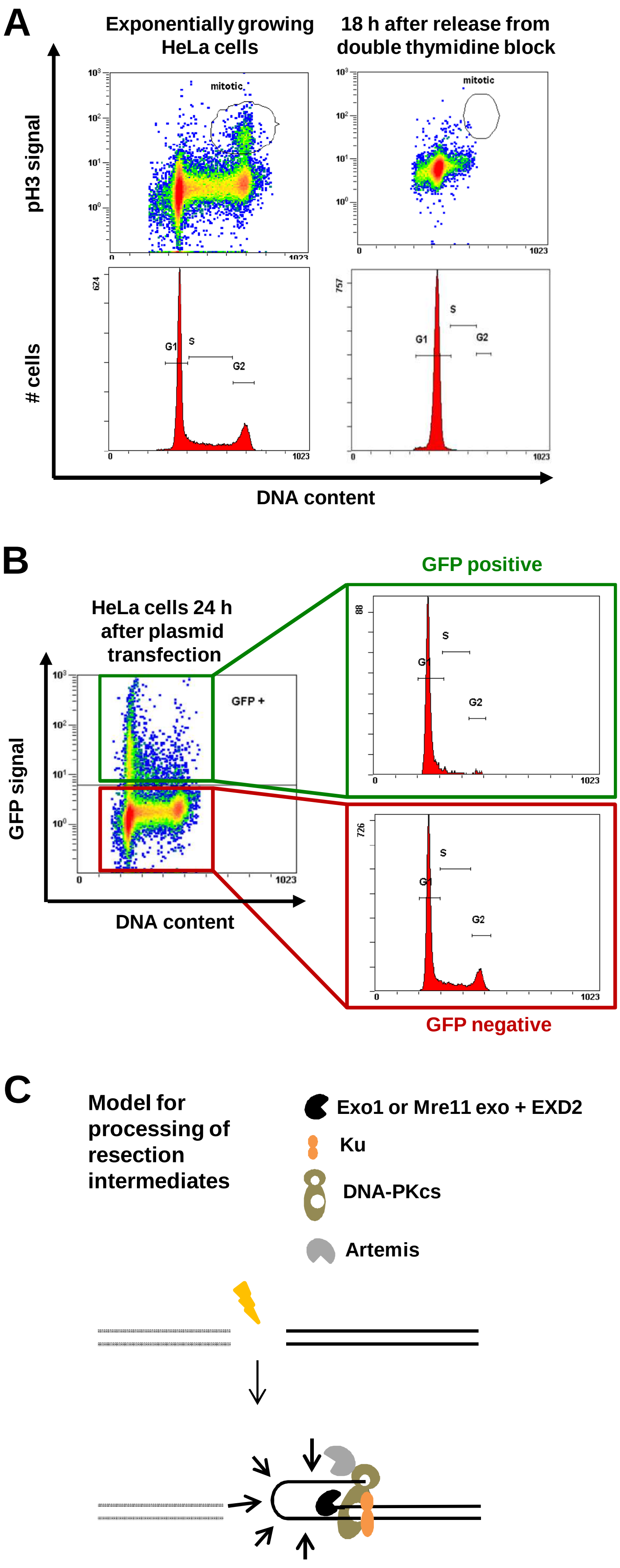
A
Exponentially growing HeLa cells
18 h after release from double thymidine block
pH3 signal
cells
mitotic
mitotic
G1
S
G2
G1
S
G2
DNA content
B
GFP positive
HeLa cells 24 h after plasmid transfection
GFP +
GFP signal
DNA content
G1
S
G2
G1
S
G2
GFP negative
C
Model for processing of resection intermediates
Exo1 or Mre11 exo + EXD2
Ku
DNA-PKcs
Artemis

SUPPLEMENTAL FIGURE LEGENDS

Figure S1. Related to Figure 1

(A) Upper images: Growing 82-6 cells were incubated with BrdU in the absence of nocodazole for 1 or 14 h before analysis by flow cytometry. Growth in the presence of BrdU (and absence of nocodazole) for 14 h results in $BrdU^+$ G1 cells and depletion of the $BrdU^-$ G2 population. Lower images: 82-6 cells were treated with nocodazole, incubated with BrdU, irradiated or not, and analyzed 14 h later. Nocodazole efficiently prevented cells from progressing into G1 as evidenced by the absence of $BrdU^+$ G1 cells and the maintenance of the $BrdU^-$ G2 population. IR resulted in efficient G1 checkpoint induction as evidenced by the absence of early- and mid-S phase cells.

(B) Growing unirradiated 82-6 cells were pulse-labeled with EdU for 30 min and analyzed by PCC. Heavily damaged chromosome structures were confined to EdU^+ S-phase cells.

(C) Growing 82-6 cells were incubated with EdU, left unirradiated, not treated with nocodazole and analyzed by PCC 14 h later. Since cells progressed through the cell cycle uninhibited, EdU^+ chromosome spreads were obtained in heavily damaged S-phase cells as well as in G1 cells with a one-chromatid and in G2 cells with a two-chromatid morphology.

(D) Growing 82-6 cells were incubated with EdU, irradiated, treated with nocodazole and analyzed by PCC 14 h later. Since nocodazole prevented progression from G2 to G1, all cells with a one-chromatid morphology were negative for EdU whilst EdU^+ cells were frequently detected in spreads with heavily damaged chromosomes and in spreads with a two-chromatid morphology. Two-chromatid G2 and mitotic cells can be easily distinguished from one-chromatid G1 cells.

(E) Growing HeLa cells were stained for the G1 marker CDT1 (upper panel) and the CDT1 signal was plotted as a function of the DAPI signal (lower panel). Only G1 cells stain positive for CDT1.

(F) Growing 82-6 cells were analyzed by PCC and stained against CDT1. The images show a mitotic HeLa cell fused with either a one-chromatid (upper row) or a two-chromatid 82-6 cell (lower row). Only the one-chromatid but not the two-chromatid PCC spread stained positive for the G1 marker CDT1 (the same was observed for all other one-chromatid and two-chromatid PCC spreads). Note that during cell rupture, which occurs while transferring the chromosomes to the glass slides, chromatin-bound CDT1 detaches from the chromosomes but remains detectable by IF staining in the nuclear area.

Figure S2. Related to Figure 1

(A) Chromosome spreads of mitotic 82-6 cells. Growing cells were incubated with nocodazole for 14 h, the mitotic cell fraction was harvested by mitotic shake-off and analyzed with or without the PCC approach. Both approaches failed to provide one-chromatid spreads while two-chromatid mitotic structures were clearly visible which typically are more condensed than the two-chromatid structures of G2 cells. This control experiment shows that mitotic 82-6 cells do not give rise to one-chromatid PCC spreads.

(B) Chromosome breaks and translocations in one-chromatid PCC spreads obtained from confluent 82-6 cells treated with DNA-PKi at 6 h post IR. The results confirm the analysis of the one-chromatid PCC spreads obtained from growing 82-6 cells in Figure 1D. Mean +/- SEM.

(C) Chromosome breaks and translocations in PCC spreads obtained from confluent 82-6 and Artemis-deficient CJ179 cells. The results confirm the analysis of the one-chromatid PCC spreads obtained from growing 82-6 and CJ179 cells in Figure 1E. Mean +/- SEM.

(D) Left panel: 82-6 cells were treated with nocodazole, incubated with BrdU, irradiated or not, and analyzed 8 h later by flow cytometry. Nocodazole efficiently prevented cells from progressing into G1 as evidenced by the absence of $BrdU^+$ G1 cells and the maintenance of the $BrdU^-$ G2 population. IR resulted in efficient G1 checkpoint induction as evidenced by the absence of early S-phase cells. Right panels: Identification of cell cycle phases using a semi-automated scanning system. Cells were incubated with nocodazole and EdU 30 min prior to IR and during the entire repair period. Cells were scanned under the microscope; EdU and GFP signals were plotted against the DAPI signal. All EdU^+ S-phase cells were excluded from the analysis and only EdU^- G1 and G2 cells, which remained in these cell cycle phases during repair incubation, were analyzed. For complementation experiments, only GFP^+ EdU^- G1 cells were analyzed.

(E) γH2AX foci in G1 HeLa cells treated with siKu80 plus PARPi or siLig1/3. Mean +/- SEM.

Figure S3. Related to Figure 2

(A) Left panel: HeLa cells were treated with nocodazole, incubated with BrdU, irradiated or not, and analyzed 8 h later by flow cytometry. Nocodazole efficiently prevented cells from progressing into G1 as evidenced by the absence of $BrdU^+$ G1 cells and the maintenance of the $BrdU^-$ G2 population. Despite the lack of an efficient G1 checkpoint, a substantial proportion of cells remained in G1 during repair incubation. Right panel: γH2AX foci in G1 HeLa cells treated with siArtemis and/or siCtIP. Mean +/- SEM.

(B) BrdU foci in G1 HeLa cells treated with siArtemis. Mean +/- SEM.

(C) γH2AX foci in G1 82-6 and CJ179 cells treated with siCtIP, PARP or DNA-PK inhibitors. Mean +/- SEM.

(D) pRPA foci in G1 HeLa cells treated with siArtemis, siCtIP, siExo1, siBrca1 or Mre11 exo- or endonuclease inhibitors. Mean +/- SEM.

(E) Ku and pRPA staining in G1 HeLa cells. Co-localization was analyzed by line blots and observed in >95% of pRPA foci. White circles: Co-localizing foci; red circles: non-co-localizing foci.

(F) Ku and Rad51 staining in G2 HeLa cells. Co-localization was analyzed by line blots and observed in <20% (after 20 Gy, 4 h) or <10% (after 4 Gy, 8 h) of Rad51 foci. White circles: Co-localizing foci; red circles: non-co-localizing foci.

Figure S4. Related to Figure 4

(A) pATM and γH2AX foci in HeLa cells treated with Mre11 endo- or exonuclease inhibitor. NT: not treated.

(B) Chromosome breaks in G1 82-6 and CJ179 cells treated with Mre11 endo- or exonuclease inhibitor, siExo1 or siBrca1. Mean +/- SEM.

(C) Relative gene conversion frequencies in HeLa pGC cells containing an HR reporter substrate (Mansour et al., 2008) and treated with Mre11 endo- or exonuclease inhibitor. Mean +/- SEM.

(D) γH2AX foci in G2 82-6 and CJ179 cells treated with Mre11 endo- or exonuclease inhibitor or siExo1. Mean +/- SEM.

(E) Knock-down efficiencies in HeLa and GC92 cells.

Figure S5. Related to Figure 5

(A) pRPA foci in G1 HeLa and end-joining events in GC92 cells treated with si53BP1. Cells were transfected with GFP, RFP or HA-53BP1-wt constructs and GFP^+, RFP^+ or HA^+ cells were analyzed. Mean +/- SEM.

(B) Left panel: End-joining events in GC92 cells treated with siLig1/3 and/or si53BP1. Right panel: γH2AX foci in GC92 cells treated with siLig1/3 and/or si53BP1. Cells were transfected with I-SceI and foci were scored in $I\text{-}SceI^+$ and $I\text{-}SceI^-$ cells (identified by IF against I-SceI). Mean +/- SEM.

(C) γH2AX foci in G1 MEFs treated with siArtemis. Cells were transfected with GFP or cMyc-Artemis-wt constructs and foci were analyzed in GFP^+ or $cMyc^+$ G1 cells. Mean +/- SEM.

(D) and (E) Brca1 and γH2AX foci in G1 and G2 HeLa cells after X-IR (panel D) or α-IR (panel E). NT: not treated.

Figure S6. Related to Figure 6

(A) γH2AX foci in G2 HeLa cells treated with siArtemis and/or siPlk3. Mean +/- SEM.

(B) γH2AX foci and chromosome breaks in G1 and/or G2 82-6 and CJ179 cells treated with siPlk3 or Plki. Mean +/- SEM.

(C) Left panel: γH2AX foci in G1 82-6 cells treated with siArtemis and/or siCtIP. Cells were transfected with GFP, GFP-CtIP-wt, a phospho-mimic (GFP-CtIP-S327E) or a non-phosphorylatable mutant (GFP-CtIP-S327A) and foci were analyzed in GFP^+ G1 cells. Right panel: IF images showing similar transfection efficiencies for the various CtIP constructs. Mean +/- SEM.

Figure S7. Related to Figure 7

(A) Flow cytometry analysis of HeLa cells growing exponentially or 18 h after release from a double thymidine block, showing synchronization in G1 phase.

(B) Flow cytometry analysis of HeLa cells 24 h after transfection with GFP-CtIP constructs. The cell cycle distribution shows G1 synchronisation of the GFP^+ cells.

(C) Speculative model for processing of resection intermediates: Ku translocates inwards and restricts the extent of resection by Exo1 or Mre11 exonuclease/ EXD2. The ssDNA tail is captured by the ssDNA channel of DNA-PKcs generating a hairpin-like structure which is cleaved by Artemis bound to DNA-PKcs (arrows indicate potential cleavage sites). In this model Artemis is required for processing of trapped resection intermediates and subsequent formation of ssDNA. pRPA foci arise when cleavage results in a ssDNA tail long enough for RPA binding (estimated to be >20 bp). DNA-PKcs may either remain loosely bound to Ku80 during the entire process or may attach to the ss/dsDNA transition at the resected end after Ku translocation but before cleavage by Artemis.

Table S1. Sequence analysis of end-joining events after Lig1/3 depletion, related to Figure 3

Amount n=33	siCtrl + I-SceI	Size of deletion	Size of insertion
6 x	C A C G G A A G G A A T T A C C C T G T T A T C C C T A T	0	0
1 x	C A C G G A A G G A A T T A C C C T G T T - - C C C T A T	2	0
1 x	C A C G G A A G G A A T T A C C C T - - - A T C C C T A T	3	0
1 x	C A C G G A A G G A A T T A - - - T G T T A T C C C T A T	3	4
1 x	C A C G G A A G G A A T T A C C C T G - - - - C C C T A T	4	0
1 x	C A C - - - - G G A A T T A C C C T G T T A T C C C T A T	4	0
1 x	C A C G G A A G G A A T T A C C C T G T T - - - - - - A T	6	0
2 x	C A C G G A A G G A A T T A - - - - - - - - T C C C T A T	8	0
6 x	C A C G G A A G G A A T T A C C C T - - - - - - - - - A T	9	0
1 x	C A C G G A A G G A A T - - - - - - - - - - - - - - T A T	14	2
1 x	C A C G G A A G G - - - - - - - - - - - - - - - C C T A T	15	6
1 x	C A - C T A T	23	7
1 x	C G C T - [-] - - - - - - - - - - - - - - - - - C T A T	34	0
1 x	C G C T - [-] - - - - - - - - - - - - - - - - - C T A T	34	3
1 x	G C G C - [-] - - - - - - - - - - - - - - - - - - T A T	36	0
1 x	C A C G - - - - - - - - - - - - - - - - - [-] - C C A T	42	0
1 x	A G A G - [-] - - - - - - - - - - - - - [-] - T C A C	44	10
1 x	C T G G - [-] - - - - - - - - - - - - - [-] - C T A G	46	13
1 x	A A T A - [-] - T	73	0
1 x	C A C G - - - - - - - - - - - - - - - - - [-] - A G C T	109	0
1 x	C A C G G A - - - - - - - - - - - - - - - [-] - G C T G	266	2
1 x	C T T G - [-] - - - - - - - - - - - - - [-] - C G G T	517	0

Amount n=25	siLig1/3 + I-SceI	Size of deletion	Size of insertion
5 x	C A C G G A A G G A A T T A C C C T G T T A T C C C T A T	0	0
1 x	C A C G G A A G G A A T T A C C C T - T T A T C C C T A T	1	0
1 x	C A C G G A A G G A A T T A C C C T - T T A T C C C T A T	1	13
1 x	C A C G G A A G G A A T T A C C C T - - - A T C C C T A T	3	0
2 x	C A C G G A A G G A A T T A - - - - - - - - T C C C T A T	8	0
1 x	C A C G G A A G G A A T T - - - - - - - - A T C C C T A T	8	0
1 x	C A C G G A A G G A A T - - - - - - - - - A T C C C T A T	9	0
1 x	C A C G G A A G G A A T T A C C C T G T - [-] - C T A G	9	0
2 x	C A C G G A A G G A A T T A C C C T - - - - - - - - - A T	9	0
1 x	C A C G G A A G G A A - - - - - - - - - - - T C C C T A T	11	0
1 x	C A C G G A A G G A A T T A C C - - - - - - - - - C T A T	13	3
1 x	C A C G G A A G G A - - - - - - - - - - - [-] - T A T G	24	0

1 x	C A C G G A A - - - - - - - - - - - - - - [-] - T C A C	34	0
1 x	T A G A - [-] - - - - - - - - - - - - - [-] - T A T G	38	0
1 x	G C T A - [-] - T	42	0
1 x	T G G C - [-] - - - - - - - - - - - - - [-] - C A T G	63	0
1 x	C C A T - [-] - - - - - - - - - - - - - - - - - - - **A** T	174	0
1 x	C A - - - - - - - - - - - - - - - - - - - [-] - G C T G	281	0
1 x	C A C G G A A G G A **A** - - - - - - - - - - [-] - C T G C	342	0

Sequence analysis of GC92 wt and siLig1/3-treated cells. The 18 nucleotide long I-SceI recognition sequence is in **bold**, the characteristic I-SceI cutting site is labeled yellow, and microhomologies are labeled red. All sequences are arranged according to their siRNA treatment and to the size of their deletion. Some sequences contained inserted nucleotides in their deleted region.

List of Insertions

siCtrl

D=3; I=4 : TTCA

D=14; I=2 : CA

D=15; I=6 : GTAATT

D=23; I=7 : AGCTGTT

D=34; I=3 : TGG

D=44; I=10 : CACAACACGG

D=46; I=13 : GTAAGCTTACAAG

D=266; I=2 : CA

siLig1/3

D=1; I=13 : AGCTAGATATGAA

D=13; I=3 : TAT

Table S2. List of sequence-based reagents used in this study, related to Star Methods

Sequence-Based Reagents		
siRNA targeting sequence: human 53BP1:<br>AGA ACG AGG AGA CGG UAA UAG UGG G	Qiagen	N/A
siRNA targeting sequence: human 53BP1:<br>GAG AGC AGA TGA TCC TTT A	Dharmacon	N/A
siRNA targeting sequence: human Artemis:<br>AAC TGA AGA GAG CTA GAA CAG	Qiagen	N/A
siRNA targeting sequence: mouse Artemis:<br>AAG GAT CAC ATG AAA GGA TTA	Qiagen	N/A
siRNA targeting sequence: human BLM:<br>AAG CTA GGA GTC TGC GTG CGA	Qiagen	N/A
siRNA targeting sequence: human Brca1:<br>AAT CAC AGT GTC CTT TAT GTA	Qiagen	N/A
siRNA targeting sequence: human CtIP1:<br>TCC ACA ACA TAA TCC TAA TAA	Qiagen	N/A
siRNA targeting sequence: human CtIP2:<br>AAG CTA AAA CAG GAA CGA ATC	Qiagen	N/A
siRNA targeting sequence: human DNA2:<br>AAA TAG CCA GTA GTA TTC GAT	Qiagen	N/A
siRNA targeting sequence: human DNA-PKcs:<br>CTC GTG TAT TAC AGA AGG AAA	Qiagen	N/A
siRNA targeting sequence: human EXD2: smart pool	Dharmacon	N/A
siRNA targeting sequence: human Exo1:<br>CAA GCC TAT TCT CGT ATT TTT	Qiagen	N/A
siRNA targeting sequence: human Ku70:<br>GGA AGA GAT AGT TTG ATT TTT	Qiagen	N/A
siRNA targeting sequence: human Ku80:<br>AAG ACA GAC ACC CTT GAA GAC	Qiagen	N/A
siRNA targeting sequence: human Lig1:<br>GGC ATG ATC CTG AAG CAG A	Qiagen	N/A
siRNA targeting sequence: human Lig3:<br>CCA CAA AAA AAA TCG AGG A	Qiagen	N/A
siRNA targeting sequence: human Lig4:<br>CAA GAT GTT TAC AGA AAG GAA	Qiagen	N/A
siRNA targeting sequence: human Plk3:<br>CTG CAT CAA GCA GGT TCA CTA	Qiagen	N/A
siRNA targeting sequence: control:<br>AAT TCT CCG AAC GTG TCA CGT	Qiagen	N/A
Artemis gRNA sequence:<br>GAG ACT TCA GAT TGG CGC A<br>GAG CCC GTA CCA TGT TGT G	This paper	N/A
PCR primer sequence: CMV2:<br>ATA TAT GGA GTT CCG CGT TAC AT	This paper	Eurofins
PCR primer sequence: CD4int:<br>GCT GCC CCA GAA TCT TCC TCT	This paper	Eurofins

SiRNA targeting sequences used for protein downregulation, gRNA sequences used for generation of CRISPR/Cas9 knockout and PCR primer sequences used for sequence analysis.
